# Genetically determined circulating resistin concentrations and risk of colorectal cancer: a two-sample Mendelian randomization study

**DOI:** 10.1007/s00432-023-05193-0

**Published:** 2023-08-21

**Authors:** Thu Thi Pham, Katharina Nimptsch, Nikos Papadimitriou, Krasimira Aleksandrova, Mazda Jenab, Marc J. Gunter, Loic Le Marchand, Li Li, Brigid M. Lynch, Sergi Castellví-Bel, Amanda I. Phipps, Stephanie L. Schmit, Hermann Brenner, Shuji Ogino, Edward Giovannucci, Tobias Pischon

**Affiliations:** 1 Molecular Epidemiology Research Group, Max Delbrueck Center for Molecular Medicine in the Helmholtz Association (MDC), 13125 Berlin, Germany; 2 Charité-Universitätsmedizin Berlin, Corporate Member of Freie Universität Berlin and Humboldt-Universität zu Berlin, 10117 Berlin, Germany; 3 Nutrition and Metabolism Branch, International Agency for Research on Cancer (IARC-WHO), World Health Organization, 150 Cours Albert Thomas, 69372 Lyon, Cedex 08, France; 4 Department of Epidemiological Methods and Etiological Research, Leibniz Institute for Prevention Research and Epidemiology—BIPS, 28359 Bremen, Germany; 5 Faculty of Human and Health Sciences, University of Bremen, 28359 Bremen, Germany; 6 Department of Epidemiology and Biostatistics, School of Public Health, Imperial College London, London, UK; 7 Epidemiology Program, University of Hawaii Cancer Center, Honolulu, HI 96813 USA; 8 Department of Family Medicine and UVA Comprehensive Cancer Center, University of Virginia, Charlottesville, Virginia USA; 9 Cancer Epidemiology Division, Cancer Council Victoria, Melbourne, Australia; 10 Centre for Epidemiology and Biostatistics, Melbourne School of Population and Global Health, The University of Melbourne, Melbourne, Australia; 11 Physical Activity Laboratory, Baker Heart and Diabetes Institute, Melbourne, Australia; 12 Department of Gastroenterology, Institut d’Investigacions Biomèdiques August Pi i Sunyer (IDIBAPS), Centro de Investigación Biomédica en Red de Enfermedades Hepáticas y Digestivas (CIBERehd), Hospital Clínic, Universitat de Barcelona, Barcelona, Spain; 13 Fred Hutchinson Cancer Center, Seattle, WA USA; 14 Genomic Medicine Institute, Cleveland Clinic, Cleveland, OH USA; 15 Population and Cancer Prevention Program, Case Comprehensive Cancer Center, Cleveland, OH USA; 16 Division of Clinical Epidemiology and Aging Research, German Cancer Research Center (DKFZ), Heidelberg, Germany; 17 Division of Preventive Oncology, German Cancer Research Center (DKFZ) and National Center for Tumor Diseases (NCT), Heidelberg, Germany; 18 German Cancer Consortium (DKTK), German Cancer Research Center (DKFZ), Heidelberg, Germany; 19 Program in Molecular Pathological Epidemiology, Department of Pathology, Brigham and Women’s Hospital and Harvard Medical School, Boston, MA USA; 20 Department of Epidemiology, Harvard T.H. Chan School of Public Health, Boston, MA USA; 21 Broad Institute of MIT and Harvard, Cambridge, MA USA; 22 Cancer Immunology and Cancer Epidemiology Programs, Dana-Farber Harvard Cancer Center, Boston, MA USA; 23 Department of Nutrition, Harvard T.H. Chan School of Public Health, Boston, MA USA; 24 Biobank Technology Platform, Max Delbrueck Center for Molecular Medicine in the Helmholtz Association (MDC), 13125 Berlin, Germany; 25 Core Facility Biobank, Berlin Institute of Health at Charité-Universitätsmedizin Berlin, 13125 Berlin, Germany

**Keywords:** Resistin, Colorectal cancer, Mendelian randomization, Genetic, Instrumental

## Abstract

**Purpose:**

Resistin, a novel pro-inflammatory protein implicated in inflammatory processes, has been suggested to play a role in colorectal development. However, evidence from observational studies has been inconsistent. Mendelian randomization may be a complementary method to examine this association.

**Methods:**

We conducted a two-sample Mendelian randomization to estimate the association between genetically determined circulating resistin concentrations and risk of colorectal cancer (CRC). Protein quantitative trait loci (pQTLs) from the SCALLOP consortium were used as instrumental variables (IVs) for resistin. CRC genetic summary data was obtained from GECCO/CORECT/CCFR (the Genetics and Epidemiology of Colorectal Cancer Consortium, Colorectal Cancer Transdisciplinary Study, and Colon Cancer Family Registry), and FinnGen (Finland Biobank). The inverse variance weighted method (IVW) was applied in the main analysis, and other robust methods were used as sensitivity analyses. Estimates for the association from the two data sources were then pooled using a meta-analysis approach.

**Results:**

Thirteen pQTLs were identified as IVs explaining together 7.80% of interindividual variation in circulating resistin concentrations. Based on MR analyses, genetically determined circulating resistin concentrations were not associated with incident CRC (pooled-IVW-OR per standard deviation of resistin, 1.01; 95% CI 0.96, 1.06; *p* = 0.67. Restricting the analyses to using IVs within or proximal to the resistin-encoding gene (*cis*-IVs), or to IVs located elsewhere in the genome (*trans*-IVs) provided similar results. The association was not altered when stratified by sex or CRC subsites.

**Conclusions:**

We found no evidence of a relationship between genetically determined circulating resistin concentrations and risk of CRC.

**Supplementary Information:**

The online version contains supplementary material available at 10.1007/s00432-023-05193-0.

## Introduction

Resistin is a protein that was first identified as an “adipose-tissue-specific secretory factor” with insulin resistance functions in mouse models (Kim et al. 2001; Steppan et al. 2001). In contrast to mice, circulating resistin in humans is secreted by macrophages, monocytes, and other peripheral blood mononuclear cells (Patel et al. 2003; Codoñer-Franch and Alonso-Iglesias 2015). In humans, it has been shown to exhibit pro-inflammatory properties upon secretion (Bokarewa et al. 2005; Zuniga et al. 2017) and to be induced by pro-inflammatory stimuli (Lehrke et al. 2004; Anderson et al. 2007), thus, plays an important role in inflammatory processes (Bokarewa et al. 2005; Codoñer-Franch and Alonso-Iglesias 2015; Zuniga et al. 2017). Since inflammation predisposes colorectal cancer (CRC) and promotes its development, resistin has been proposed as a molecule that may be linked to cancer development (Tuomisto et al. 2019). Case–control studies suggest that resistin levels measured in CRC patients (i.e., post-diagnosis) are higher compared to controls (Yang et al. 2016). In contrast, two prospective cohort studies found no statistically significant association between pre-diagnostic resistin concentrations and CRC risk (Ho et al. 2012; Pham et al. 2022). The Women’s Health Initiative (WHI) reported a relative risk (RR) of 1.04 (95% CI 0.72, 1.50) of CRC among postmenopausal women when comparing the highest versus lowest quartile of resistin concentrations (Ho et al. 2012). Based on data from the European Investigation into Cancer and Nutrition (EPIC) study, we have previously observed a RR of 1.15 (95% CI 0.91, 1.46) (Pham et al. 2022). However, both studies relied on a single measurement of resistin concentrations per individual for exposure assessment. Although resistin concentrations have shown to be relatively reliable over 3–4 years in human bodies (Kaplan et al. 2007; Weikert et al. 2007), a single measurement may still be subjected to within-person variation, which may attenuate RR estimates of exposure-disease associations towards the null (White 2011). Therefore, the overall picture of the role of resistin for CRC risk remains unclear, especially given that based on these previous studies small effects cannot be ruled out. Genetic variations in the resistin gene’s promoter were found to strongly influence plasma resistin concentrations in humans (Cho et al. 2004). Thus, a 2-sample Mendelian randomization (MR) study based on large-sample-size genome-wide association studies (GWAS) may be an alternative robust method providing adequate power to estimate this association.

A MR study uses germline genetic variants that are robustly and independently associated with levels of a protein (so-called protein quantitative trait loci (pQTLs)) as instrumental variables (IVs) for the protein to estimate the association with an outcome (Burgess and Thompson 2015). MR analyses are conventionally used to reduce the risk of residual confounding or reverse causality that may occur in observational studies (Wehby et al. 2008; Burgess et al. 2013, 2019; Burgess and Thompson 2015). Due to the random assortment of alleles occurring during gamete formation, genetically increased resistin concentrations are a phenotype that always occurs before CRC diagnosis (Burgess et al. 2019) or any potentially confounding factors, thus, limiting the possibility of reverse causation and residual confounding and eliminating the influence of differential bias. Determining the association between genetically elevated resistin concentrations and risk of CRC in fact also reduces the impact of non-differential misclassification due to within-person variation in resistin concentrations. Despite the advantages of MR analysis, so far, no study using genetic variants as IVs in the relationship between resistin and risk of CRC has been conducted. Herein, we implemented an instrumental variable approach to estimate the quantitative association between genetically determined resistin concentrations and risk of CRC using two-sample Mendelian randomization (Supplementary Fig. 1).

## Materials and methods

### Study design: two-sample Mendelian randomization

**Fig. 1:**
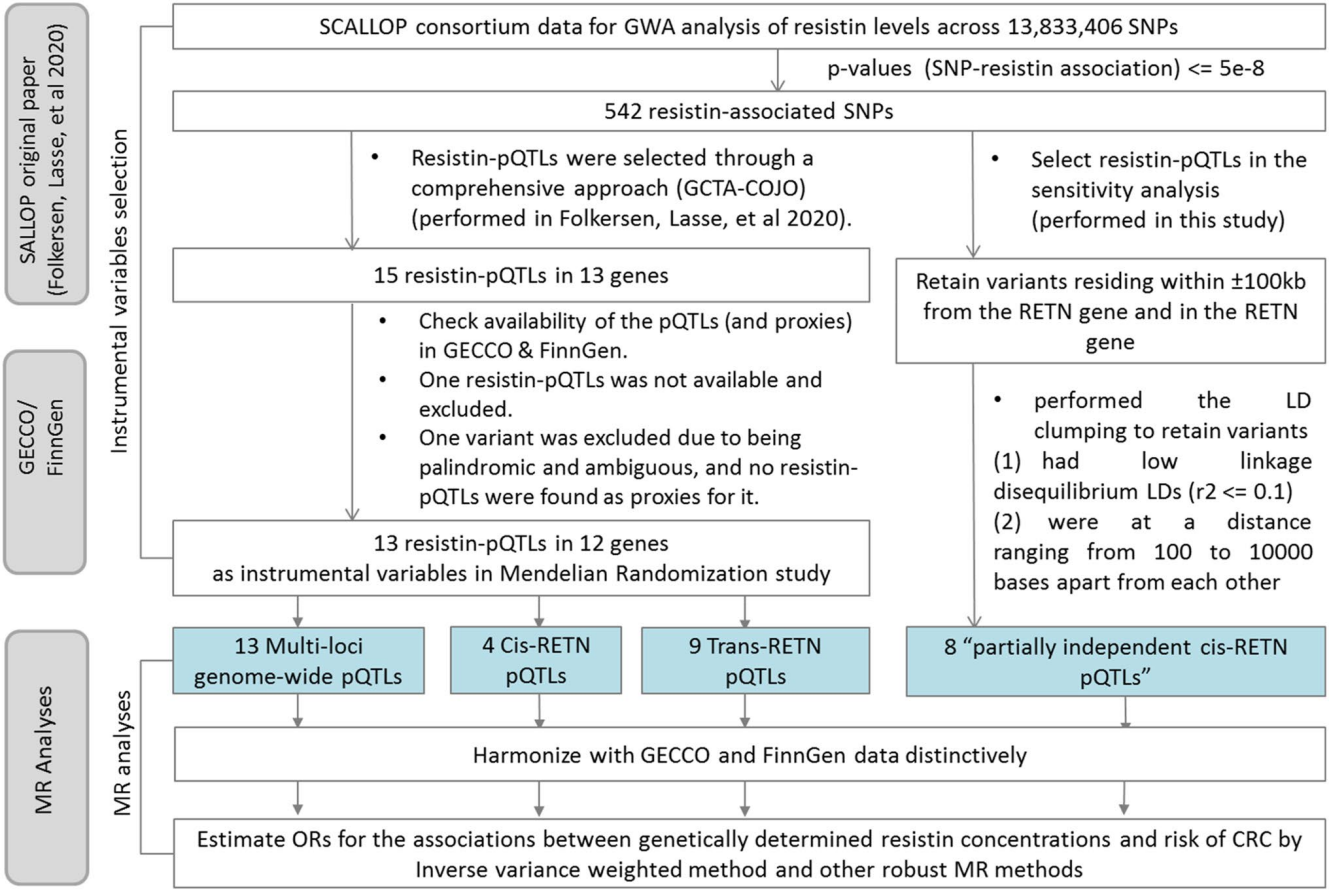
Workflows describing instrumental variable selection and MR analyses. *GWA* genome-wide association, *pQTLs* protein quantitative trait loci, *SCALLOP* Systematic and Combined AnaLysis of Olink Proteins consortium, *GECCO* Genetics and Epidemiology of Colorectal Cancer Consortium, *FinnGen* Finnish biobanks

A two-sample MR study was performed using published GWAS summary statistics on the association of pQTLs with resistin concentrations from the Systematic and Combined Analysis of Olink Proteins (SCALLOP consortium) (Folkersen et al. 2020) and GWAS summary statistics on CRC risk from the Genetics and Epidemiology of Colorectal Cancer Consortium (GECCO), Colorectal Cancer Transdisciplinary Study (CORECT), and Colon Cancer Family Registry (CCFR), hereafter, the studies will be collectively referred to as GECCO for simplicity (Huyghe et al. 2019, 2021), and the Finnish biobank (FinnGen, publicly accessible and downloadable) (Kurki et al. 2022). A graphical illustration of conceptual MR models describing the relationship between IVs, resistin, and CRC is described in Supplementary Fig. 2 and a workflow describing IVs selection and MR analyses is in Fig. 1.

### GWAS summary statistics on resistin concentrations (exposure data)

The SCALLOP consortium is a collaborative framework to map pQTLs and analysis biomarker data on the Olink platform (Folkersen et al. 2020). The consortium is an ongoing project with an increasing number of joining research institutions and cohort studies (https://www.olink.com/our-community/scallop/). The most recently published SCALLOP study harmonized the summary statistics of Olink CVD-I protein data in a total of 21,758 individuals from 13 GWASs (Folkersen et al. 2020). Among contributing GWAS, 9 studies were population-based studies, one randomized control trial (RCT) in blood donors, one RCT in chronic coronary heart disease, one study in metabolic syndrome patients, and one Bipolar cases-control study (Folkersen et al. 2020) (detailed description of GWAS in SCALLOP are provided in the in Supplement). SCALLOP provides meta-analysis estimates of summary-level data on 27 million gene variants and 90 CVD-I proteins (including resistin) that were derivable for secondary analyses (available at GWAS catalog and fully available at 10.5281/zenodo.2615265 for unrestricted download access) (Folkersen et al. 2020). The mapping of resistin-pQTLs was described in the original publication (Folkersen et al. 2020). Briefly, summary statistics of 13.8 million imputed autosomal variants and resistin levels had been available in 20,471 individuals of European descent (Folkersen et al. 2020). At the conventional *p* value threshold for GWAS (*p* < 5 × 10^−8^), 542 SNPs had been found to be significantly associated with resistin concentrations. These significantly associated SNPs had mutual high linkage disequilibrium (LD), and using all of them as IVs in a MR analysis would have resulted in false positive estimations (Burgess et al. 2017). An approximate conditional and joint analysis using GCTA-COJO (genome-wide complex trait conditional and joint analysis) software had been performed to retain lead SNPs for each independent locus as primary signals (9 SNPs), and then additionally select independent SNPs by a stepwise procedure as secondary signal (6 SNPs) (Folkersen et al. 2020). Finally, a total of 15 genetic variants which were robustly and independently associated with resistin levels (also referred to as pQTLs) had been identified and referred to as pQTLs (Folkersen et al. 2020). We retrieved summary statistic data of these 15 pQTLs (rs199752470, rs17405635, rs7589428, rs6775731, rs2239619, rs7746716, rs73008259, rs77691416, rs445, rs10103048, rs3087852, rs4134826, rs3745367, rs34861192, and rs10401670) from the original publication (Folkersen et al. 2020).

### GWAS summary statistics on colorectal cancer (outcome data)

Summary statistics of 15 selected IVs and risk of CRC risk were extracted from two datasets, including the GECCO consortium (Huyghe et al. 2019, 2021) and the FinnGen consortium (Finland Biobank, R7, downloaded on 18/07/2022) (Kurki et al. 2022).

GECCO is the largest and most comprehensive GWAS meta-analysis for CRC to date, consisting of 45 GWAS studies, resulting in a total of 58,131 CRC cases (31,083 colon; 13,857 proximal; 15,306 distal; and 15,775 rectal cancer cases) and 67,347 controls (Huyghe et al. 2019, 2021). In each contributing GWAS, genome-wide CRC association analyses had been performed using logistic regression models adjusted for study-defined principal components, age, gender, and study-specific covariates (Huyghe et al. 2019). The estimates from all GWAS had been combined using a fixed-effect meta-analysis. We requested meta-analysis summary statistics of the associations between 15 resistin-pQTLs (or their proxies) and CRC risk used in the current MR study from GECCO.

Finngen is a research project combining imputed genotype data integrated from Finnish biobanks and digital health registry records (Kurki et al. 2022). The R7 release of FinnGen has a total sample size of 309,154 (173,746 women and 135,408 men (https://www.finngen.fi/en/access_results), among them, 4957 individuals developed CRC (2989 colon cancer, 1832 rectal cancer, and 136 undefined or rectosigmoid junction). GWAS analyses had been carried out using the mixed model logistic regression method SAIGE, adjusted for gender, age, genotyping batch, and the first 10 genetically derived principal components as covariates (Kurki et al. 2022). Summary statistics of all associations estimated by the FinnGen GWAS had then be made publicly available for download. We check the availability of all resistin pQTLs in the summary data of CRC retrieved from FinnGen R7 release. When data of a pQTLs were not available in the summary statistics of CRC data, we replaced it with proxies by using LDlinkR (https://github.com/CBIIT/LDlinkR/) which outputs information on all variants $\pm$ 500 Kb of the query variant (Myers et al. 2020) to find a genetic variant with a correlation coefficient (*r*^2^) greater than 0.80 for those IVs. Where more than one proxy was found for the index pQTLs, one genetic variant with the highest *r*^2^ was selected.

### Final data set, *cis*-pQTLs, and *trans*-pQTLs

Among 15 resistin-pQTLs, the pQTL rs199752470 was not available in GECCO and FinnGen and no proxies were found for this variant, thus, it was excluded from the MR analysis. The resistin-pQTL rs73008259 was accessible in GECCO; however, it was not available in FinnGen and was substituted with rs72992130 in FinnGen (LD-R2 = 0.80, distance at + 30.33 Kb of the index variant). Furthermore, rs7746716 was a palindromic and ambiguous variant (defined as a variant with the same pair of bases on the forward and reverse strands and minor allele frequency (MAF) close to 0.5 (MAF of rs7746716 = 0.48) and no resistin-associated variants could be found as proxy at LD-R2 ≥ 0.8 for it, and thus, was excluded. Eventually, the summary statistics of 13 pQTLs were derived from GECCO and FinnGen and used as final IVs in the main analysis (Table 1). We classified SNPs into two groups based on their locations as follows: *cis* variants including those resided within 1 megabase upstream or downstream of the transcription start site of the resistin-coding gene (RETN gene) and *trans* variants including those located elsewhere in the genome (Melzer et al. 2008). These 13 pQTLs are located widely in 8 different chromosomes, among those, 4 are *cis*-RETN pQTLs, and 9 pQTLs are *trans*-RETN pQTLs. Top pQTLs associated with resistin concentrations included three *cis*-pQTLs (rs3745367, rs10401670, and rs34861192). Among 13 resistin-pQTLs (Table 1), no pQTLs were statistically significantly associated with CRC in GECCO data, while 2 *trans*-pQTLs (rs7589428, and rs2239619) were significantly associated with CRC in FinnGen data (Supplementary Fig. 2).

**Table 1:** Summary statistics of the association of genetic variants as instrumental variables with circulating resistin concentrations in the SCALLOP consortium

No.	SNP	Chr	Position	Gene closest	*cis* or *trans* ^b^	Effect allele	Ref allele	EAF	Effect	SE	*p* value	Sample size	Proportion of variance explained	*F*-statistic
1	rs17405635	2	43355763	ZFP36L2	*trans*	A	G	0.26	0.08	0.011	6.6e−14	20793	0.002	41.7
2	rs7589428	2	43561771	THADA	*trans*	A	G	0.51	0.063	0.0095	4.6 e−11	21747	0.002	43.6
3	rs6775731	3	128306894	RPN1	*trans*	T	C	0.3	− 0.063	0.011	3.2 e−09	21747	0.002	43.6
4	rs2239619	6	52453220	TRAM2	*trans*	A	C	0.62	− 0.053	0.0097	4.1 e−08	21749	0.001	21.8
5	rs77691416	6	144354119	PLAGL1	*trans*	A	C	0.91	0.12	0.016	7.9 e−14	18353	0.002	36.8
6	rs73008259 ^a^	6	144411338	SF3B5	*trans*	A	G	0.053	0.19	0.02	3.7 e−21	16199	0.004	65.0
7	rs445	7	92408370	CDK6	*trans*	T	C	0.1	− 0.085	0.015	3.3 e−08	18353	0.001	18.4
8	rs10103048	8	130602281	GSDMC	*trans*	A	C	0.42	0.06	0.0096	5.2 e−10	21747	0.002	43.6
9	rs3087852	17	38137364	PSMD3	*trans*	A	G	0.46	− 0.086	0.0094	6.6 e−20	21749	0.004	87.3
10	rs34861192	19	7733575	RETN	*cis*	A	G	0.017	1.10	0.054	7.8 e−89	15136	0.040	630.6
11	rs3745367	19	7734511	RETN	*cis*	A	G	0.24	0.12	0.012	7.5 e−23	18353	0.005	92.2
12	rs10401670	19	7742802	MCEMP1	*cis*	T	C	0.43	0.15	0.012	9 e−37	16221	0.011	180.4
13	rs4134826	19	7692076	XAB2	*cis*	T	C	0.86	− 0.089	0.014	2.6 e−10	18353	0.002	36.8

Data of resistin protein quantitative trait loci (resistin-pQTLs) were retrieved from the original publication of the SCALLOP consortium (Folkersen et al 2020) and were available for retrieving from GECCO or FinnGen. Effect sizes (linear regression coefficients) are given as per standard deviation of protein concentrations per effect allele. At the conventional *p* value threshold for GWAS (*p* < 5 × 10^−8^), 542 SNPs were significantly associated with resistin concentrations. Only 15 SNPs were retained as resistin-pQTLs which are robustly and independently associated with levels of resistin. Two SNPs were excluded from the analyses, including one SNP that was not available in both GECCO and FinnGen, and one SNP was palindromic with ambiguous strand identification exhibiting an intermediate minor allele frequency (MAF = 0.48). Eventually, 13 resistin-pQTLs were selected as IVs for the MR analysis

^a^Data of rs73008259 is not available in FinnGen and was replaced by rs72992130 (LD-r2 = 0.82, distance at + 30.33 Kb of the index variant)

^b^*cis* pQTLs including variants residing within 1 megabase upstream or downstream of the transcription start site of the resistin-encoded gene (RETN gene) and tran pQTLs including variants residing elsewhere in the genome

*Chr* chromosome, *Ref allele* reference allele, *EAF* effect allele frequency, *SE* standard error of the effect

### Mendelian randomization analysis

*F*-statistics for each pQTL and mean *F*-statistic for all pQTLs together were estimated using the formula *F*-statistic = ((*N*−*K*−1)/*K*) × (*R*^2^/(1−*R*^2^))) where *R*^2^ is the proportion of variance in the exposure explained by the genetic variants, *N* is the sample size and *K* is the number of instruments (Burgess and Thompson 2015). Variance explained by each pQTL was calculated using the formula *R*^2^ = 2 × (Beta*X*)^2^ × EAF × (1 − EAF) (Burgess and Thompson 2015) where Beta*X* is the genetic association with resistin concentrations [expressed as standard deviation (SD) units], and EAF is the effect allele frequency. Given that the genetic variants serving as IVs in this analysis are uncorrelated, the total proportion of variance explained by all IVs is the sum of variance explained by each IV. Weak instruments were considered variants with an *F*-statistic below 10 (Burgess et al. 2013). Furthermore, we estimated the minimum detectable ORs of the MR analyses by using a webtool at https://sb452.shinyapps.io/power/ (Brion et al. 2013). Of note, the number of controls excluding other cancers used in the power estimation in the MR using FinnGen data was 245,442, which was obtained based on the number of CRC cases, the unadjusted prevalence, and the total number of cancer cases, and (https://r7.risteys.finngen.fi/phenocode/C3_COLORECTAL; accessed on 18/07/2022).

The association between genetically determined circulating resistin concentrations and risk of CRC was estimated using all 13 variants as IVs by multiplicative random-effect inverse variance weighted (IVW) method with the assumption that all variants were valid IVs and the mean pleiotropic effect is zero (“balanced pleiotropy”). Estimates from the IVW method were derived from weighted linear regression of the genetic associations with CRC risk on the genetic associations with resistin levels with a zero intercept (Bowden et al. 2015). Results for the relationship between genetically determined circulating resistin concentrations and risk of CRC are reported as odds ratios per SD unit increase of genetically predicted resistin (OR_per SD of resistin_) and 95% confidence intervals (CIs). MR analyses were further carried out for *cis*-pQTLs and *trans*-pQTLs. The association was estimated separately for SCALLOP-GECCO and SCALLOP-FinnGen and pooled using a meta-analysis approach by a random effects model with an inverse variance method to assign weight given to each IV (Borenstein et al. 2010). Several sensitivity analyses were conducted to detect and correct for horizontal pleiotropy if present. First, we conducted MR analyses using other methods to complement the results from the main IVW analysis. The MR-Egger method was performed to allow the genetic variants partially influence the risk of CRC through different causal pathways (“horizontal pleiotropy”) by introducing an intercept in the regression of the genetic associations with CRC risk on the genetic associations with resistin levels (Bowden et al. 2015). Furthermore, simple median, weighted median, and weighted mode methods were applied to relax the pleiotropy assumption on 50% of genetic variants by estimating point estimates as the unweighted/weighted median (Burgess et al. 2017) or the mode of the smoothed empirical Kernel density function of the estimates (Hartwig et al. 2017) by each variant individually. Second, we retrieved summary statistic data of all 542 resistin-associated SNPs and performed the LD clumping to retain variants (“the partially independent *cis*-pQTLs”) which (i) reside within ± 100 kb from the RETN gene and in the RETN gene, (ii) had low linkage disequilibrium LDs (*r*^2^ < = 0.1) and, (iii) were at a distance ranging from 100 to 10,000 bases apart from each other. Next, we conducted a MR analysis accounting for the correlation matrix among these variants. Third, we re-estimated the effect by sequentially dropping one pQTL at a time (leave-one-variant-out analysis) to evaluate if the MR estimate is driven or biased by a single pQTL that might have a particularly large horizontal pleiotropic effect. Fourth, we extracted and aligned all possible phenotypes of all 13 resistin-pQTLs from GWAS Catalog using phenoscanner::phenoscanner() function in R, and performed a MR analysis after removing SNPs that may influence CRC developments through other pathways rather elevated resistin levels. In subgroup analyses, we stratified by sex, and we analyzed CRC sites separately (colon, proximal colon, distal colon, and rectal cancer).

The presence of pleiotropy manifested as heterogeneity among IVs was examined by Cochran’s *Q* and *I*^2^ statistics in a meta-analysis of MR estimates (Bowden et al. 2015, Greco et al. 2015) and by *p* values of the MR Egger regression intercept tests with null hypotheses that the intercepts are equal to zero, and a value of *p* < 0.05 suggests the presence of directional pleiotropy (Bowden et al. 2015). Cochran’s *Q* follows a chi-square distribution with *n* − 1 degrees of freedom when pleiotropy absents, and *I*^2^ is the percentage of the total variation of the estimates explained by heterogeneity rather than sampling errors (Bowden et al. 2015). A funnel plot of MR estimates for each pQTL against its precision was provided to assess potential asymmetry referred to as “directional” pleiotropy (Bowden et al. 2015).

All analyses were carried out in R version 4.0.5 (R Foundation for Statistical Computing). Data wrangling and manipulation were implemented using the tidyverse package, and data harmonization and MR analysis were performed using the *TwoSampleMR* and *MendelianRandomization* R packages.

## Results

The proportion of variance explained by the 13 pQTLs selected as IVs was 7.80% (the *cis*-pQTLs alone explained 5.80% of the variance). The mean *F*-statistic for the IVs used for genetically determined circulating resistin levels was 103.2, and the *F*-statistics of individual variants were all higher than 10 declining the presence of weak instruments (Table 1). With 80% power, using data from GECCO (sample size: 125,478; the ratio of cases to controls: 0.86) and FinnGen (sample size: 250,399; the ratio of cases to controls: 0.0198) as outcome data, the minimum detectable for ORs per SD from the two datasets were 1.06 and 1.16, respectively.

**Fig. 2:**
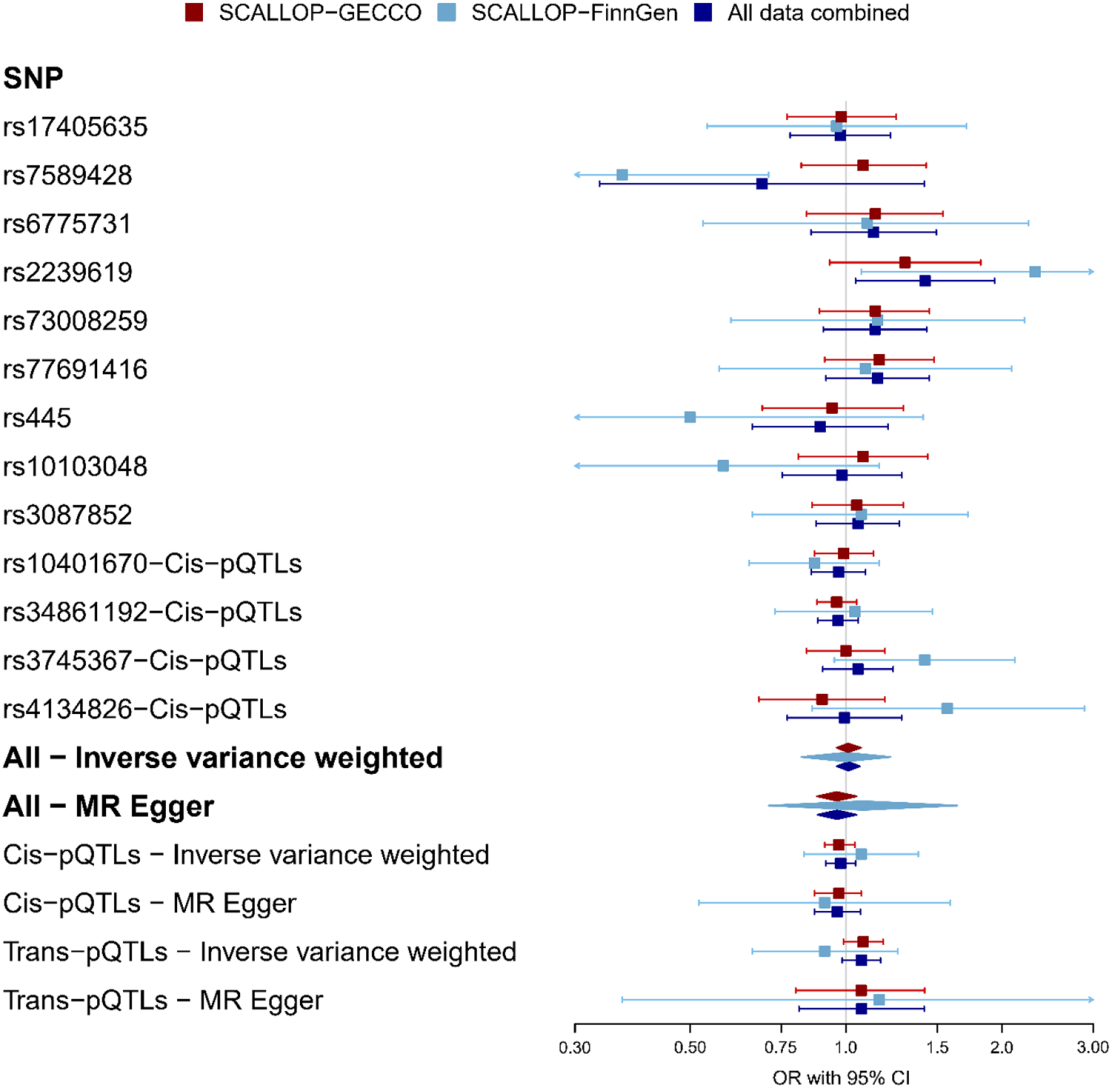
Two-sample Mendelian randomization analysis of the relationship between genetically determined circulating resistin concentrations and risk of colorectal cancer estimated using each individual IV, *cis*-pQTLs, *trans*-pQTLs, and multi-loci genome-wide IVs. ORs (95% CI) estimated for the effect of one SD increase in genetically predicted serum resistin concentrations and risk of CRC. MR analyses were performed separately for GECCO and FinnGen, and combined using a meta-analysis approach by random effects models with inverse variance method (results are in “All data combined”). Quantifying heterogeneity among 13 SNPs in the combined estimates: *τ*^2^ (Sidik–Jonkman estimator for between-study variance in a random-effects meta-analysis) = 0.0096 [0.0000; 0.0272]; *I*^2^ (total variability in a set of effect sizes due to true heterogeneity) = 0.0% [0.0%; 56.6%]. Test of heterogeneity: *Q* = 9.11, *p* = 0.69. A *cis*-pQTL was defined as a pQTL residing within 1 megabase (Mb) upstream or downstream of the transcription start site of the corresponding protein-coding gene; in this figure, “rs34861192”, “rs4134826”, “rs3745367”, and “rs10401670” are resistin *cis*-pQTL. A *trans*-pQTL was defined as a pQTL residing elsewhere in the genome. Data of rs73008259 is not available in FinnGen and was replaced by rs72992130 (LD-r2 = 0.82, distance at + 30.33 Kb of the index variant)

A scatter plot of the 13 pQTLs‐resistin against the pQTLs‐CRC risk associations along with their 95% CIs is shown in Supplementary Fig. 2. Point estimates from the MR analyses by IVW and MR-Egger methods are shown in Fig. 2 and Supplementary Fig. 2. The IVW estimates indicated that there is no statistically significant association between genetically determined resistin concentrations and risk of CRC using GECCO data (OR_per SD of resistin_, 1.01; 95% CI 0.96, 1.07; *p* = 0.67) or using FinnGen data (OR_per SD of resistin_, 1.00; 95% CI 0.82, 1.22; *p* = 0.99) or all data sources combined (OR_per SD of resistin_, 1.01; 95% CI 0.96, 1.06; *p* = 0.67) (Fig. 2, Supplementary Fig. 2). Point estimates from MR-Egger method were not different from those from IVW method suggesting no significant association and tests of MR-Egger regression zero-intercepts suggested no evidence of directional pleiotropy (*p* values were 0.12 and 0.70 for GECCO and FinnGen, respectively) (Fig. 2, Supplementary Table 1). Similar results to the IVW estimates were provided by the simple median, weighted median, and weighted mode methods (Supplementary Table 1). We also found no significant association between genetically determined resistin concentrations and risk of CRC when restricting the analysis to *cis*-QTLs or *trans*-QTLs and applying the IVW method (IVW-OR_per SD of resistin, *cis*-QTLs_, 0.98 (95% CI 0.92, 1.04) and IVW-OR_per SD of resistin, *trans*-QTLs_, 1.07 (95% CI 0.98, 1.17), or other robust methods (e.g. results from the MR Egger method were not different from the IVW estimates and all *p* values for intercepts of the MR-Egger regression > 0.05) (Fig. 2, Supplementary Table 1). In the MR analysis using single-variant, we found a significant association between resistin and CRC risk when only the *trans*-pQTL rs2239619 was used as an IV, but not other pQTLs (Fig. 2).In the sensitivity analysis, by using “the partially independent *cis*-pQTLs”, we included eight variants including rs10401670, rs2161490, rs34124816, rs35547567, rs3745367, rs4804766, rs62110711, and rs77509849 as IVs. MR analyses using these variants accounted for the matrix of correlations between variants showing an IVW-OR of 0.97 (95% CI 0.91, 1.04) and an MR-Egger OR of 0.95 (95% CI 0.85, 1.06) with the p values of MR Egger regression intercept of 0.60) (Supplementary Table 1). Furthermore, leave-one-variant-out analyses showed that the main results were not changed even after excluding one variant each time and performing MR analyses using the remaining 12 variants or the remaining 3 *cis*-pQTLs or the remaining 8 *trans*-pQTLs (Supplementary Table 2). Phenotypes of all variants extracted and aligned from GWAS Catalog databases using Phenoscanner are shown in Supplementary Table 3. The most frequent phenotypes of the selected pQTLs were related to the numbers or percentages of lymphocyte, monocyte, neutrophil, basophil, eosinophil, granulocyte, myeloid white cell, and white blood cell—the most important features of inflammation, although there was one pQTL (rs2239619) with phenotypes as low-density lipoprotein (LDL) or total cholesterol. MR analysis using only this variant resulted in a significant association (OR estimated by rs2239619, 1.42; 95% CI 1.04, 1.94; *p* = 0.03) (Fig. 2); however, dropping this variant did not change the main results (OR estimated from all SNPs excluding rs2239619, 1.00; 95% CI 0.95, 1.06; *p* = 0.91 (Supplementary Table 2). There was no evidence of heterogeneity between the pQTLs used as IVs for circulating resistin concentrations in IVW estimate when using GECCO data (Cochran’s *Q* for IVW *p* = 0.82, *I*^2^ = 0%). However, heterogeneity was present in FinnGen data with a Cochran’s *Q* *p* = 0.02 and *I*^2^ = 42%). An approximately symmetric distribution in the funnel plot was observed (Supplementary Fig. 3). No statistically significant association of genetically determined resistin concentrations and risk was observed when stratifying by sex or when analyzing CRC subsites separately (colon, proximal, distal, rectum) (Table 2, Supplementary Table 1).

**Table 2:** Two-sample Mendelian randomization analysis of the relationship between genetically determined circulating resistin concentrations and risk of colorectal cancer by subsite and gender

Subgroup	OR (95% CI)^a^	*p* value
Subsite—all SNPs		
Colon^b^	0.98 (0.91, 1.05)	0.54
Rectal^b^	1.01 (0.93, 1.09)	0.89
Proximal colon^c^	0.93 (0.84, 1.03)	0.14
Distal colon^c^	1.02 (0.93, 1.11)	0.68
Subsite—*cis*-RETN		
Colon^b^	0.95 (0.87, 1.04)	0.24
Rectal^b^	0.95 (0.85, 1.06)	0.38
Proximal colon^c^	0.94 (0.80, 1.10)	0.46
Distal colon^c^	0.94 (0.84, 1.06)	0.30
Gender—all SNPs		
Female^c^	0.98 (0.91, 1.06)	0.59
Male^c^	1.05 (0.97, 1.13)	0.25
Gender—*cis*-RETN		
Female^c^	0.94 (0.85, 1.04)	0.21
Male^c^	1.01 (0.91, 1.11)	0.88

^a^ORs and 95% CIs estimated for the effect of 1-standard deviation genetically determined higher serum resistin concentrations on risk of CRC from inverse variance weighted models

^b^Estimates from a meta-analysis of estimates derived from GECCO and FinnGen data

^c^Data were not available in FinnGen, thus, estimates were derived from GECCO only

## Discussion

We found no statistically significant association between genetically determined circulating resistin concentrations and risk of CRC among individuals of European descent in this two-sample Mendelian randomization study using 13 pQTLs as IVs for resistin concentrations. Robust analysis methods with different sets of IVs including all pQTLs, *cis*-pQTLs, *trans* pQTLs, or “partially independent *cis*-pQTLs” revealed no significant association of interest. Subgroup analyses showed that genetically determined circulating resistin concentrations were not associated with risk of CRC subsites regardless of whether the analysis was stratified by sex or not. Conclusively, no associations between genetically predicted resistin levels were observed despite high statistical power to detect even weak associations.

To our knowledge, this is the first two-sample MR study investigating the relationship between genetically determined resistin concentrations and CRC risk using statistical genetic approaches via summary statistics. In line with our MR study, findings from two traditional observational biomarker studies suggested no association between pre-diagnosis resistin concentrations and CRC or any subsite of CRC risk (Ho et al. 2012; Pham et al. 2022). Of note, our previous prospective study had estimated relative risks of CRC per doubling resistin concentrations (Pham et al. 2022) which were not totally comparable to the current MR study (OR_per SD of resistin_). We, therefore, re-estimated the RR for per SD unit increase of resistin concentrations for our previous prospective observational study and appraised an RR of 1.03 (95% CI 0.94, 1.12; *p* = 0.54) which indicated no significant association. Furthermore, it is notable that in our previous prospective study, we found a significant association of circulating resistin concentrations with risk of CRC in participants diagnosed within 2 years after blood sampling and their matched controls [RR 4th vs 1st quartile of resistin concentrations, 1.97 (95% CI 1.06, 3.64)], whereas no significant association was found in participants diagnosed with CRC after more than 2 years of blood sampling and their matched controls [RR 4th vs 1st quartile of resistin concentrations, 1.44 (95% CI 0.97, 2.12)] (Pham et al. 2022). Case–control studies, in which resistin concentrations were measured after CRC cancer diagnosis, reported higher resistin concentrations in CRC patients as compared to controls (Nakajima et al. 2010; Danese et al. 2012) which may be due to inflammation in the presence of the existing CRC tumors. These observations, therefore, suggest that resistin is not related to risk of CRC and that increased resistin concentrations observed in people who were diagnosed with CRC shortly after recruitment are likely the result of the existing, but yet undiagnosed tumors, i.e. possibility of reverse causation. However, the previous biomarker studies used only a one-time measurement of resistin concentrations (Ho et al. 2012; Pham et al. 2022) and may neglect the small effect due to within-person variability (White 2011). Alternatively, in our current MR study we were able to investigate the association between genetically determined lifelong differences in circulating resistin in relation to CRC risk (Burgess et al. 2019) that was less susceptible to attenuation due to within-individual variations of resistin levels (Pierce and VanderWeele 2012) and circumvented reverse causation (Burgess et al. 2019). Furthermore, multiple loci from different gene regions (Burgess et al. 2019) were employed in this MR study as IVs provide high statistical power to identify the small effect sizes. Importantly, results from analyses that used *cis*-pQTLs or “partially independent *cis*-pQTLs” which were commonly referred to as “biologically plausible instrumental variables” (Burgess et al. 2019) complemented the main findings and indicated no significant association. Of note, *cis*-pQTLs are variants located within or in close proximity to the RETN gene, are thus expected to have a direct effect on resistin expression. Thereby, *cis*-pQTLs could provide precise and targeted measures of resistin levels by RETN gene expression. Conclusively, our MR analysis is generally in agreement with the two prospective observational studies, does not support the hypothesis that circulating resistin concentrations are associated to risk of CRC, and suggests that resistin is a marker of existing tumors rather than a causal risk factor.

Although pleiotropy is a major concern in MR, we do not suspect that it is a significant issue in this study. Indeed, a series of sensitivity analyses were conducted in the present study, including robust MR analysis methods that allow different patterns of pleiotropic assumption violations, different sets of IVs, leave-one-variant-out analysis, and removing potential pleiotropic variants all resulted in effect estimates close to one and suggested that the main findings in this study are reliable and robust. The pleiotropy may present in FinGenn according to Cochran’s *Q* test and the *I*^2^ index, however, these values tend to be conservative in a small sample of cases as in FinnGen (Greco et al. 2015). Furthermore, we observed no strong pattern of asymmetry in the funnel plot and thus, inferred no explicit indication of unbalanced or directional pleiotropy. Nevertheless, there is evidence pointing out that the *trans*-pQTL rs2239619 may play as a pleiotropic variant. This variant was related to LDL and total cholesterol (Spracklen et al. 2017) while studies suggest that dyslipidemia is related to a higher risk of CRC (Yao and Tian 2015), suggesting that the association of this variant with CRC risk could be explained by their involvement in dyslipidemia rather than their involvement in resistin concentrations. While a significant association was found between genetically determined resistin levels and risk of CRC when using *trans*-pQTL rs2239619 as a single-variant in the MR analysis, the main findings did not show any changes after eliminating this variant, indicating that it may not be a potentially influential variant. Thus, *trans*-pQTL rs2239619 even if exhibits pleiotropic effects, is not considered a significant issue.

The current MR study had several strengths. The associations between pQTLs-resistin were estimated in the SCALLOP consortium which is the largest-to-date meta-analysis GWAS of resistin concentrations for a maximum power of the analysis. Indeed, our study has a power of 80% for a minimum detectable OR_per SD of resistin_ of 1.06, which is ambitious in observational studies (our previous prospective observational study could provide a minimum detectable OR_per SD of resistin_ of 1.12). There is no overlap between the two samples in the current MR analysis (e.g. UK biobank was only included in the GECCO consortium), while overlapping samples may inflate the type-1 error rate and bias the estimates toward the null (Burgess et al. 2016). Furthermore, all variants used in the MR study are non-weak instrumental variables (*F* values for all individual variants > 10) (Burgess et al. 2016). We retrieved more than one data source of genetic summary data on CRC risk to investigate the robustness and the replicability of the estimates.

There are some limitations in this study. First, the study estimated the association of genetically determined resistin concentrations and risk of CRC in ethnically homogeneous participants of European descent; thus, the generalizability of the study results could only be applied to the European populations. Indeed, there may be ethnic differences in resistin-pQTLs between Asian and Caucasian populations (Kumar et al. 2019). For example, two pQTLs found as resistin-pQTLs in the Asian population (rs3219175 and rs34861192) (Asano et al. 2009; Onuma et al. 2010) were not significantly associated with resistin levels in Caucasians (Hivert et al. 2009). Second, all 13 IVs selected in this MR study merely explained 7.80% of the variance of resistin concentrations across individuals. The original paper of the SCALLOP consortium may use a more stringent discovery GWAS threshold considering the multiple testing problems when performing analyses for 90 proteins (Folkersen et al. 2020). One may question if including more variants as IVs in the MR analysis could benefit from increased variance explained while false positive rates could be controlled. Indeed, the total variation explained may be underestimated if only the lead SNPs for each independent region are selected (Yang et al. 2012). However, in SCALLOP, resistin-pQTLs were selected through a comprehensive approach (GCTA-COJO) to not only identify the lead SNPs of each region but also secondary independent association signals at a region (Folkersen et al. 2020). The GCTA-COJO approach has been shown to result in the number of pQTLs that well characterize the number of causal variants, and resulted in false-positive rates that are close to expectation (0.05) under the IVW method (van der Graaf et al. 2020). Thus, the selection of resistin-pQTLs in the SCALLOP consortium has assured the balance of variance explained and false positive rate. Furthermore, results did not change by using “the partially independent *cis*-pQTLs” set of IVs which were selected by a different approach (LD clumping) rather than the GCTA-COJO. Therefore, the selection of IVs in this study is deemed justified.

In conclusion, our study does not support the hypothesis that circulating resistin concentrations are associated with risk of CRC. Future studies should target resistin as a marker of existing tumors rather than a causal risk factor of CRC.

## Supplementary Information

Below is the link to the electronic supplementary material.

Supplementary file1 (DOCX 267 KB)

Supplementary file2 (DOCX 36 KB)

## Data Availability

Summary level data from the SCALLOP consortium are available in supplementary materials of the original publication (Folkersen et al. 2020), and FinnGen summary data are publicly available for download upon registration to gain access to FinnGen GWAS summary statistics at https://elomake.helsinki.fi/lomakkeet/102575/lomake.html, data named finngen_R7_C3_COLORECTAL_EXALLC.gz with “EXALLC” as suffixes indicated that individuals with other cancer are excluded from controls). Data from GECCO and FinnGen used in this MR study are available in supplementary materials of this paper (Supplementary Table 4).

## Abbreviations

MR: Mendelian randomization
CRC: Colorectal cancer
pQTLs: Protein quantitative trait loci
SCALLOP: The Systematic and Combined Analysis of Olink Proteins
GECCO: The Genetics and Epidemiology of Colorectal Cancer Consortium
CORECT: Colorectal Cancer Transdisciplinary Study
CCFR: Colon Cancer Family Registry
FinnGen: Finland Biobank
IVW: Inverse variance weighted
EPIC: The European Investigation into Cancer and Nutrition
WHI: Women’s Health Initiative
RR: Relative risk
OR: Odds ratios
GWAS: Genome-wide association studies
IV: Instrumental variable
GCTA-COJO: Genome-wide complex trait conditional and joint analysis
MAF: Minor allele frequency
EAF: Effect allele frequency
RETN gene: Resistin-coding gene
SD: Standard deviation
