# Supplementary material for: Genetically determined circulating resistin concentrations and risk of colorectal cancer: a two-sample Mendelian randomization study
Source: J Cancer Res Clin Oncol. 2023 Aug 21;149(16):14889–900. doi: 10.1007/s00432-023-05193-0 (PMC10602946; doi:10.1007/s00432-023-05193-0)
Supplement: Supplementary file 1 — Supplementary file1 (DOCX 267 KB) [file 432_2023_5193_MOESM1_ESM.docx]

**Title: Genetically determined circulating resistin concentrations and risk of colorectal cancer: a two-sample Mendelian Randomization study**

**Supplementary**

**Studies in the Systematic and Combined Analysis of Olink Proteins consortium (SCALLOP consortium)**

The contributing GWAS cohort was described in a supplementary file of the original publication individuals (Folkersen, Gustafsson et al. 2020). Briefly, 9 over 13 GWAS are population-based studies, one randomized control trial (RTC) in blood donors, one RCT in chronic coronary heart disease, one study in metabolic syndrome patients, and one Bipolar cases-control study. All contributing studies were in the European ancestry population except for one RCT-the Stabilization of Atherosclerotic Plaque by Initiation of Darapladib Therapy trial (STABILITY)- recruited participants worldwide with 78% reported with white as an ethnical group. Sample sizes of the contributing GWAS ranged from 496 to 4987 participants. Six among 13 GWAS were from Sweden and contributed data on 6526 participants. Other studies were all in European participants, including a study in the Netherlands (LifeLines/ LifeLines-DEEP study, n= 1178), multi-center studies in European countries (Carotid Intima Media Thickness [IMT] and IMT-Progression as Predictors of Vascular Events in a High Risk European Population study (the IMPROVE study), n= 3403; the INTERVAL study, n= 498), Scotland (n= 971), Estonia (n= 496). The STABILITY trial contributed data of 2967 participants. All the GWAS include a wide range of age, INTERVAL and LifeLines/LifeLines-DEEP studies enrolled participants aged 18 years and older, the EpiHealth study recruited participants aged from 45-75, while most of the participants from other studies had a range of age 55–80 years.

Names of 13 GWAS in the SCALOPP consortium are as follows:

1. IMPROVE: Carotid Intima Media Thickness [IMT] and IMT-Progression as Predictors of Vascular Events in a High Risk European Population study
2. SWEBIC: the Sweden Bipolar Disorder Cohort study
3. EpiHealth; Epidemiology for Health study
4. PIVUS: The Prospective Investigation of the Vasculature in Uppsala Seniors study
5. ULSAM: The Uppsala Longitudinal Study of Adult Men study
6. INTERVAL: The INTERVAL study
7. LifeLines/LifeLines-DEEP: The Prospective Cohort Study
8. NSPHS: The Northern Sweden Population Health Study
9. STABILITY: the Stabilization of Atherosclerotic Plaque by Initiation of Darapladib Therapy trial
10. Estonian BB: Estonian Biobank Project
11. ORCADES: The Orkney Complex Disease Study
12. VIS/or CROATIA-Vis: Vis (a population-based study during 2003 and 2004 in the Dalmatian island of Vis)
13. MPP-RES: The Malmö Preventive Project

Supplementary Fig. 1. Conceptual Mendelian Randomization (MR) model, with three assumptions required and methods to estimate the association of circulating resistin concentrations (X) and risk of colorectal cancer (Y).

Supplementary Fig. 2: Two-sample Mendelian randomization analysis for the associations between genetically determined circulating resistin concentrations and risk of colorectal cancer association by using genetic variants as instrumental variables

Supplementary Table 1: MR estimates of the association between genetically determined circulating resistin concentrations and risk of colorectal cancer & other subsites, and stratifying by sex

Supplementary Table 2: Leave-one instrumental variable out and effect estimates of the relationship between genetically determined circulating resistin concentrations and risk of colorectal cancer using Mendelian Randomization with inverse variance-weighted method.

Supplementary Table 3: Traits of each resistin-pQTL (in addition to resistin concentrations)

Supplementary Fig. 3: Funnel plot of MR estimates of the exposure-outcome against their precision for each exposure-outcome dataset.

Supplementary Table 4: Genetic summary data for CRC

Supplementary Fig. 1. Conceptual Mendelian Randomization (MR) model, with three assumptions required and methods to estimate the association of circulating resistin concentrations (X) and risk of colorectal cancer (Y).


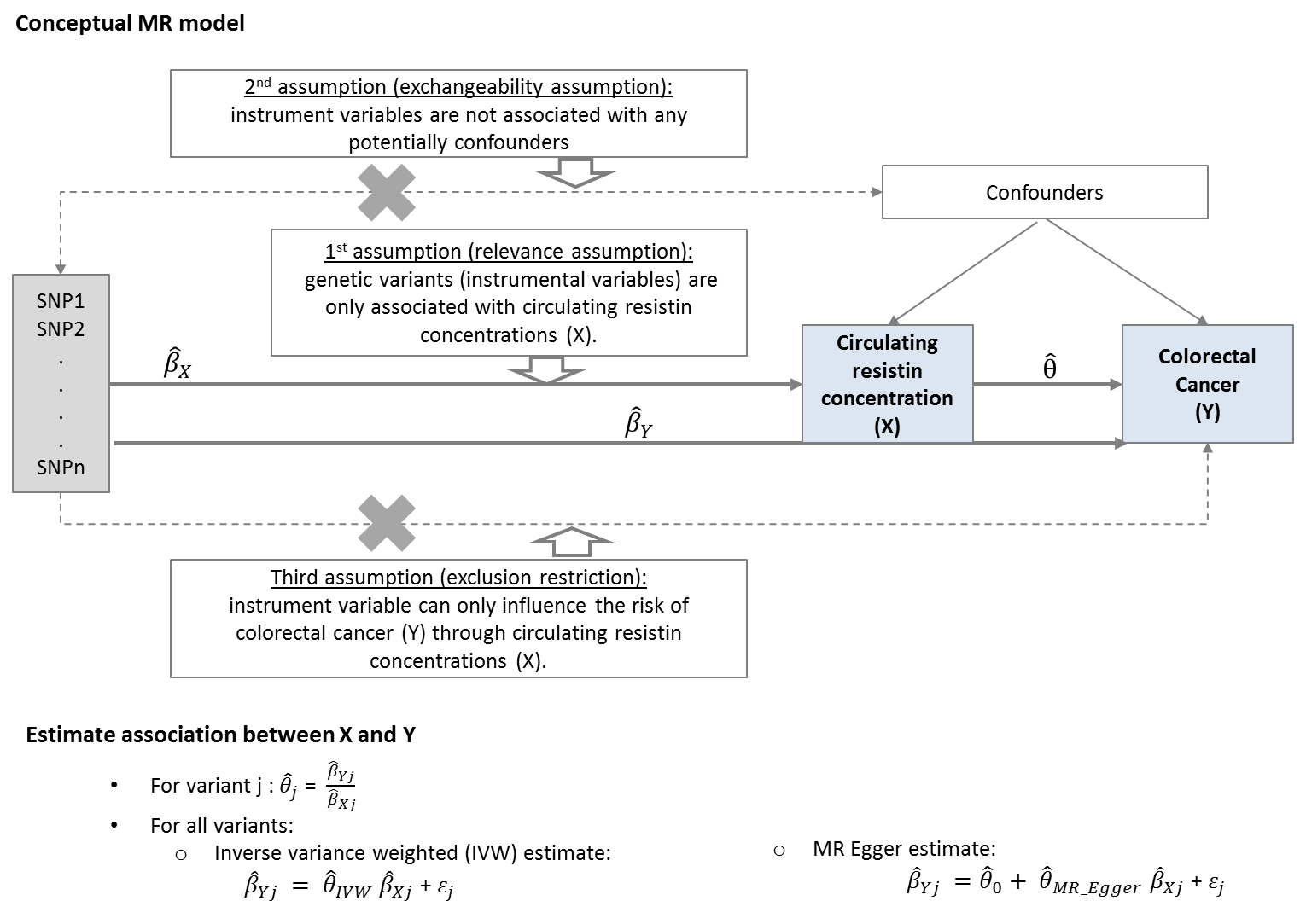


Supplementary Fig. 2: Two-sample Mendelian randomization analysis for the associations between genetically determined circulating resistin concentrations and risk of colorectal cancer association by using genetic variants as instrumental variables

| 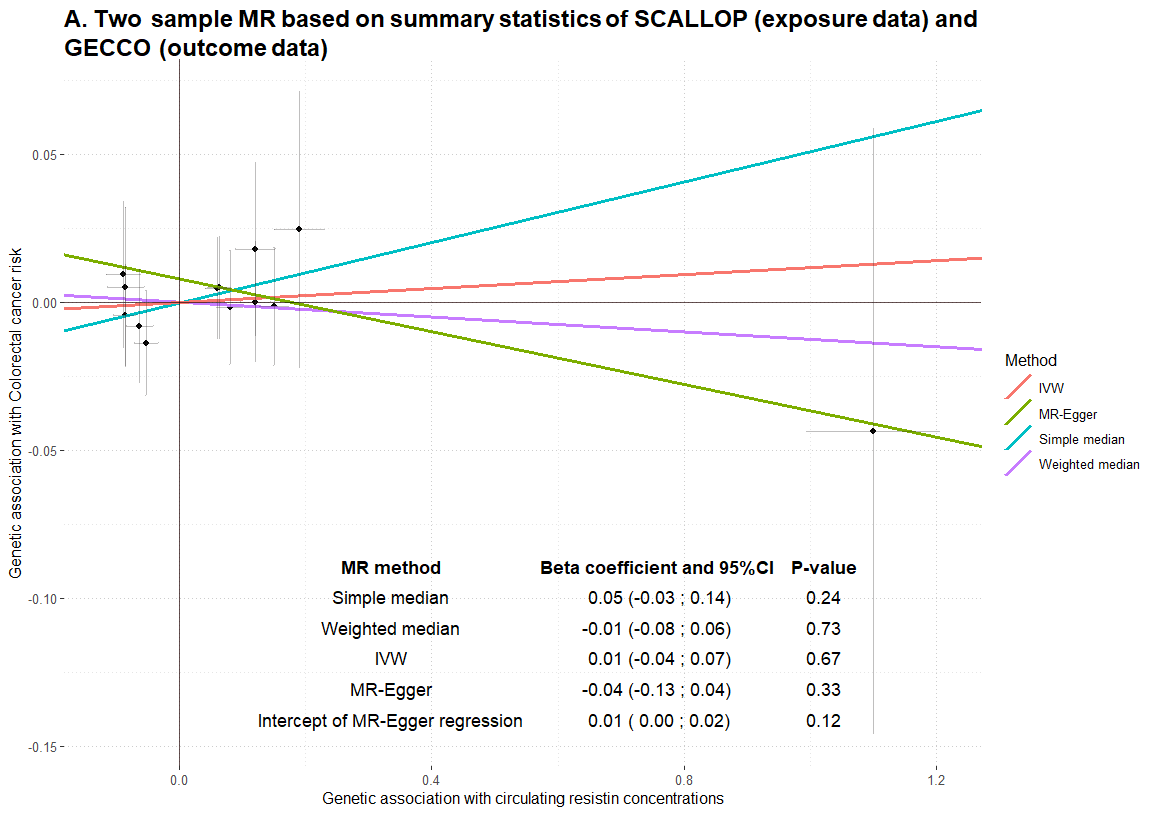 | 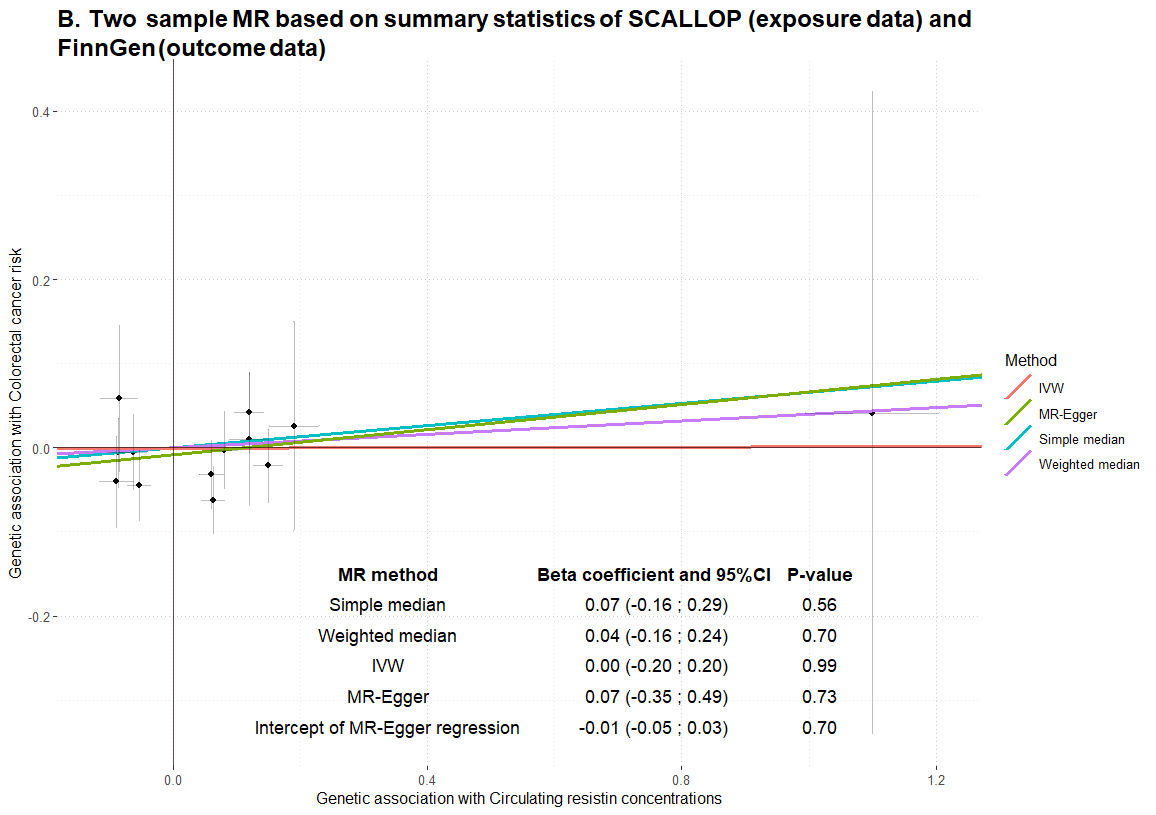 |
| --- | --- |

Each point on the plots represents one SNP. The crosshairs on the plots represent the 95% confidence intervals (CIs) for each SNP-Resistin (X-axis) vs. SNP-CRC risk association (Y-axis). The estimated effects for the association of genetically determined resistin and risk of CRC are represented by regression lines (see labels) by four models. Odd ratios and 95%CIs for the association could be estimated by exponentiating the estimates of slopes’ beta coefficients and 95%CIs. The analytical concepts of the four models were described in detail in the current manuscript. The IVW and weighted median methods with a zero intercept treated each as a valid IV and assumed that the mean pleiotropic effect is zero (“balanced pleiotropy”) and MR-Egger method relaxed the “balanced pleiotropy” assumption by introducing intercept to the model. Abbreviation: IVW: Inverse variance weighted (here, multiplicative random effects).

Supplementary Table 1: MR estimates of the association between genetically determined circulating resistin concentrations and risk of colorectal cancer & other subsites, and stratifying by sex.

| Sub-group analysis | | MR Method | SCALLOP & GECCO | | SCALLOP & FinnGen | | Pooled Estimate | |  |  |
| --- | --- | --- | --- | --- | --- | --- | --- | --- | --- | --- |
|  |  |  | OR (95%CI) | p-value | OR (95%CI) | p-value | OR (95%CI) | p-value |  |  |
| **All genome-wide pQTL as IVs** | | | | | | | | | | |
|  | CRC | Inverse variance weighted | 1.01 (0.96 , 1.07) | 0.67 | 1.00 (0.82 , 1.22) | 0.99 | 1.01 (0.96 , 1.06) | 0.67 |  |  |
|  | CRC | MR Egger | 0.96 (0.88 , 1.05) | 0.35 | 1.08 (0.71 , 1.64) | 0.73 | 0.96 (0.88 , 1.05) | 0.38 |  |  |
|  | CRC | Simple median | 1.05 (0.97 , 1.15) | 0.24 | 1.07 (0.86 , 1.33) | 0.55 | 1.05 (0.97 , 1.14) | 0.19 |  |  |
|  | CRC | Weighted median | 0.99 (0.92 , 1.06) | 0.72 | 1.04 (0.85 , 1.27) | 0.70 | 0.99 (0.93 , 1.06) | 0.84 |  |  |
|  | CRC | Weighted mode | 0.98 (0.91 , 1.06) | 0.61 | 1.01 (0.79 , 1.28) | 0.96 | 0.98 (0.91 , 1.06) | 0.63 |  |  |
|  | Colon | Inverse variance weighted | 0.97 (0.90 , 1.05) | 0.46 | 1.03 (0.83 , 1.27) | 0.80 | 0.98 (0.91 , 1.05) | 0.54 |  |  |
|  | Colon | MR Egger | 0.89 (0.79 , 1.00) | 0.08 | 1.10 (0.70 , 1.72) | 0.69 | 0.90 (0.80 , 1.01) | 0.08 |  |  |
|  | Colon | Simple median | 0.99 (0.89 , 1.11) | 0.91 | 1.11 (0.86 , 1.43) | 0.43 | 1.01 (0.92 , 1.11) | 0.84 |  |  |
|  | Colon | Weighted median | 0.99 (0.90 , 1.09) | 0.88 | 1.07 (0.84 , 1.36) | 0.59 | 1.00 (0.92 , 1.10) | 0.95 |  |  |
|  | Colon | Weighted mode | 1.00 (0.89 , 1.13) | 0.96 | 1.08 (0.83 , 1.42) | 0.57 | 1.02 (0.91 , 1.13) | 0.78 |  |  |
|  | Rectal | Inverse variance weighted | 1.01 (0.93 , 1.10) | 0.79 | 0.93 (0.68 , 1.27) | 0.65 | 1.01 (0.93 , 1.09) | 0.89 |  |  |
|  | Rectal | MR Egger | 0.93 (0.79 , 1.08) | 0.34 | 1.12 (0.59 , 2.15) | 0.73 | 0.93 (0.81 , 1.08) | 0.37 |  |  |
|  | Rectal | Simple median | 1.09 (0.95 , 1.25) | 0.24 | 0.88 (0.61 , 1.26) | 0.47 | 1.06 (0.93 , 1.20) | 0.40 |  |  |
|  | Rectal | Weighted median | 0.98 (0.87 , 1.10) | 0.74 | 0.88 (0.63 , 1.23) | 0.47 | 0.97 (0.87 , 1.08) | 0.58 |  |  |
|  | Rectal | Weighted mode | 0.96 (0.83 , 1.10) | 0.56 | 0.89 (0.59 , 1.34) | 0.58 | 0.95 (0.83 , 1.08) | 0.45 |  |  |
|  | Proximal colon cancer | Inverse variance weighted | 0.93 (0.84 , 1.03) | 0.14 | NA | NA | NA | NA |  |  |
|  | Proximal colon cancer | MR Egger | 0.89 (0.74 , 1.07) | 0.25 | NA | NA | NA | NA |  |  |
|  | Proximal colon cancer | Simple median | 0.85 (0.74 , 0.99) | 0.04 | NA | NA | NA | NA |  |  |
|  | Proximal colon cancer | Weighted median | 0.86 (0.75 , 0.97) | 0.02 | NA | NA | NA | NA |  |  |
|  | Proximal colon cancer | Weighted mode | 0.87 (0.73 , 1.04) | 0.15 | NA | NA | NA | NA |  |  |
|  | Distal colon cancer | Inverse variance weighted | 1.02 (0.93 , 1.11) | 0.68 | NA | NA | NA | NA |  |  |
|  | Distal colon cancer | MR Egger | 0.93 (0.80 , 1.09) | 0.41 | NA | NA | NA | NA |  |  |
|  | Distal colon cancer | Simple median | 1.06 (0.92 , 1.22) | 0.43 | NA | NA | NA | NA |  |  |
|  | Distal colon cancer | Weighted median | 1.01 (0.89 , 1.14) | 0.93 | NA | NA | NA | NA |  |  |
|  | Distal colon cancer | Weighted mode | 0.97 (0.84 , 1.13) | 0.74 | NA | NA | NA | NA |  |  |
| **Cis-RETN-pQTLs as IVs** | | | | | | | | | | |
|  | CRC | Inverse variance weighted | 0.97 (0.91 , 1.04) | 0.39 | 1.07 (0.83 , 1.38) | 0.59 | 0.98 (0.92 , 1.04) | 0.49 |  |  |
|  | CRC | MR Egger | 0.97 (0.87 , 1.07) | 0.57 | 0.91 (0.52 , 1.59) | 0.77 | 0.96 (0.87 , 1.07) | 0.47 |  |  |
|  | CRC | Simple median | 0.98 (0.90 , 1.06) | 0.56 | 1.21 (0.95 , 1.56) | 0.12 | 1.00 (0.92 , 1.08) | 0.95 |  |  |
|  | CRC | Weighted median | 0.97 (0.90 , 1.05) | 0.50 | 1.01 (0.79 , 1.29) | 0.95 | 0.98 (0.91 , 1.05) | 0.54 |  |  |
|  | CRC | Weighted mode | 0.97 (0.90 , 1.05) | 0.53 | 0.95 (0.72 , 1.24) | 0.71 | 0.97 (0.90 , 1.05) | 0.42 |  |  |
|  | Colon | Inverse variance weighted | 0.93 (0.85 , 1.02) | 0.14 | 1.07 (0.84 , 1.35) | 0.60 | 0.95 (0.87 , 1.04) | 0.24 |  |  |
|  | Colon | MR Egger | 0.88 (0.74 , 1.03) | 0.25 | 1.03 (0.64 , 1.66) | 0.91 | 0.89 (0.76 , 1.04) | 0.15 |  |  |
|  | Colon | Simple median | 0.93 (0.83 , 1.03) | 0.18 | 1.16 (0.87 , 1.56) | 0.31 | 0.95 (0.86 , 1.06) | 0.36 |  |  |
|  | Colon | Weighted median | 0.94 (0.84 , 1.04) | 0.23 | 1.08 (0.81 , 1.42) | 0.61 | 0.95 (0.86 , 1.05) | 0.34 |  |  |
|  | Colon | Weighted mode | 0.92 (0.80 , 1.06) | 0.32 | 0.91 (0.65 , 1.29) | 0.65 | 0.92 (0.81 , 1.05) | 0.20 |  |  |
|  | Rectal | Inverse variance weighted | 0.95 (0.85 , 1.06) | 0.34 | 1.05 (0.66 , 1.65) | 0.84 | 0.95 (0.85 , 1.06) | 0.38 |  |  |
|  | Rectal | MR Egger | 0.93 (0.78 , 1.12) | 0.54 | 0.70 (0.28 , 1.76) | 0.53 | 0.92 (0.78 , 1.10) | 0.39 |  |  |
|  | Rectal | Simple median | 0.97 (0.84 , 1.11) | 0.63 | 1.19 (0.81 , 1.76) | 0.38 | 0.99 (0.87 , 1.13) | 0.87 |  |  |
|  | Rectal | Weighted median | 0.95 (0.84 , 1.08) | 0.45 | 0.88 (0.60 , 1.30) | 0.52 | 0.94 (0.84 , 1.07) | 0.36 |  |  |
|  | Rectal | Weighted mode | 0.95 (0.83 , 1.10) | 0.54 | 0.86 (0.57 , 1.30) | 0.54 | 0.94 (0.82 , 1.08) | 0.38 |  |  |
|  | Proximal colon cancer | Inverse variance weighted | 0.94 (0.80 , 1.10) | 0.46 | NA | NA | NA | NA |  |  |
|  | Proximal colon cancer | MR Egger | 0.86 (0.65 , 1.14) | 0.41 | NA | NA | NA | NA |  |  |
|  | Proximal colon cancer | Simple median | 0.95 (0.82 , 1.10) | 0.49 | NA | NA | NA | NA |  |  |
|  | Proximal colon cancer | Weighted median | 0.99 (0.85 , 1.15) | 0.91 | NA | NA | NA | NA |  |  |
|  | Proximal colon cancer | Weighted mode | 0.99 (0.81 , 1.22) | 0.95 | NA | NA | NA | NA |  |  |
|  | Distal colon cancer | Inverse variance weighted | 0.94 (0.84 , 1.06) | 0.30 | NA | NA | NA | NA |  |  |
|  | Distal colon cancer | MR Egger | 0.95 (0.79 , 1.15) | 0.67 | NA | NA | NA | NA |  |  |
|  | Distal colon cancer | Simple median | 0.94 (0.81 , 1.09) | 0.41 | NA | NA | NA | NA |  |  |
|  | Distal colon cancer | Weighted median | 0.95 (0.83 , 1.08) | 0.43 | NA | NA | NA | NA |  |  |
|  | Distal colon cancer | Weighted mode | 0.96 (0.82 , 1.12) | 0.65 | NA | NA | NA | NA |  |  |
| **Trans-pQTLs as IVs** | | | | | | | | | | |
|  | CRC | Inverse variance weighted | 1.08 (0.99 , 1.18) | 0.07 | 0.91 (0.66 , 1.26) | 0.58 | 1.07 (0.98 , 1.17) | 0.11 |  |  |
|  | CRC | MR Egger | 1.07 (0.80 , 1.42) | 0.67 | 1.16 (0.37 , 3.66) | 0.81 | 1.07 (0.81 , 1.42) | 0.62 |  |  |
|  | CRC | Simple median | 1.08 (0.96 , 1.21) | 0.18 | 1.07 (0.78 , 1.47) | 0.68 | 1.08 (0.97 , 1.20) | 0.16 |  |  |
|  | CRC | Weighted median | 1.08 (0.97 , 1.20) | 0.16 | 1.07 (0.78 , 1.47) | 0.68 | 1.08 (0.97 , 1.19) | 0.14 |  |  |
|  | CRC | Weighted mode | 1.10 (0.93 , 1.29) | 0.30 | 1.07 (0.75 , 1.53) | 0.70 | 1.09 (0.94 , 1.27) | 0.24 |  |  |
|  | Colon | Inverse variance weighted | 1.04 (0.93 , 1.16) | 0.53 | 0.98 (0.67 , 1.43) | 0.91 | 1.03 (0.93 , 1.15) | 0.57 |  |  |
|  | Colon | MR Egger | 0.97 (0.67 , 1.42) | 0.90 | 1.14 (0.29 , 4.48) | 0.86 | 0.99 (0.68 , 1.42) | 0.94 |  |  |
|  | Colon | Simple median | 1.00 (0.87 , 1.15) | 0.96 | 1.03 (0.68 , 1.57) | 0.89 | 1.01 (0.88 , 1.15) | 0.93 |  |  |
|  | Colon | Weighted median | 1.00 (0.87 , 1.15) | 0.99 | 1.07 (0.74 , 1.55) | 0.72 | 1.01 (0.89 , 1.15) | 0.89 |  |  |
|  | Colon | Weighted mode | 1.00 (0.82 , 1.20) | 0.96 | 1.14 (0.69 , 1.89) | 0.63 | 1.01 (0.85 , 1.21) | 0.90 |  |  |
|  | Rectal | Inverse variance weighted | 1.12 (0.97 , 1.28) | 0.11 | 0.79 (0.50 , 1.25) | 0.31 | 1.09 (0.95 , 1.24) | 0.22 |  |  |
|  | Rectal | MR Egger | 1.11 (0.71 , 1.74) | 0.67 | 2.11 (0.48 , 9.32) | 0.36 | 1.17 (0.76 , 1.80) | 0.47 |  |  |
|  | Rectal | Simple median | 1.10 (0.91 , 1.33) | 0.32 | 0.62 (0.35 , 1.11) | 0.11 | 1.04 (0.87 , 1.25) | 0.66 |  |  |
|  | Rectal | Weighted median | 1.10 (0.91 , 1.32) | 0.31 | 0.79 (0.47 , 1.33) | 0.38 | 1.06 (0.89 , 1.26) | 0.51 |  |  |
|  | Rectal | Weighted mode | 1.15 (0.88 , 1.50) | 0.35 | 0.48 (0.19 , 1.19) | 0.15 | 1.07 (0.82 , 1.38) | 0.62 |  |  |
|  | Proximal colon cancer | Inverse variance weighted | 0.91 (0.78 , 1.06) | 0.21 | NA | NA | NA | NA |  |  |
|  | Proximal colon cancer | MR Egger | 0.80 (0.47 , 1.36) | 0.44 | NA | NA | NA | NA |  |  |
|  | Proximal colon cancer | Simple median | 0.85 (0.71 , 1.03) | 0.10 | NA | NA | NA | NA |  |  |
|  | Proximal colon cancer | Weighted median | 0.86 (0.72 , 1.02) | 0.09 | NA | NA | NA | NA |  |  |
|  | Proximal colon cancer | Weighted mode | 0.85 (0.68 , 1.07) | 0.20 | NA | NA | NA | NA |  |  |
|  | Distal colon cancer | Inverse variance weighted | 1.14 (1.00 , 1.31) | 0.06 | NA | NA | NA | NA |  |  |
|  | Distal colon cancer | MR Egger | 1.18 (0.75 , 1.86) | 0.49 | NA | NA | NA | NA |  |  |
|  | Distal colon cancer | Simple median | 1.21 (1.00 , 1.46) | 0.05 | NA | NA | NA | NA |  |  |
|  | Distal colon cancer | Weighted median | 1.21 (1.02 , 1.44) | 0.03 | NA | NA | NA | NA |  |  |
|  | Distal colon cancer | Weighted mode | 1.22 (0.96 , 1.56) | 0.14 | NA | NA | NA | NA |  |  |
| **“The partially independent cis-pQTLs” as IVs *** | | | | | | | | | |  |
|  | CRC | IVW | 0.96 (0.89 , 1.03) | 0.29 | 1.13 (0.89 , 1.43) | 0.32 | 0.97 (0.91 , 1.04) | 0.46 |  |  |
|  | CRC | Egger | 0.92 (0.82 , 1.03) | 0.14 | 1.39 (0.94 , 2.04) | 0.10 | 0.95 (0.85 , 1.06) | 0.34 |  |  |
|  | Colon cancer | IVW | 0.94 (0.86 , 1.03) | 0.16 | 1.08 (0.86 , 1.36) | 0.50 | 0.96 (0.88 , 1.04) | 0.29 |  |  |
|  | Colon cancer | Egger | 0.91 (0.79 , 1.05) | 0.18 | 1.42 (0.97 , 2.10) | 0.07 | 1.04 (0.80 , 1.34) | 0.78 |  |  |
|  | Rectal cancer | IVW | 0.97 (0.87 , 1.09) | 0.65 | 1.25 (0.86 , 1.82) | 0.24 | 0.99 (0.89 , 1.11) | 0.92 |  |  |
|  | Rectal cancer | Egger | 0.90 (0.75 , 1.07) | 0.23 | 1.48 (0.76 , 2.89) | 0.25 | 0.93 (0.78 , 1.10) | 0.39 |  |  |
|  | Proximal colon cancer | IVW | 0.94 (0.83 , 1.06) | 0.32 | NA | NA | NA | NA |  |  |
|  | Proximal colon cancer | Egger | 0.93 (0.75 , 1.13) | 0.46 | NA | NA | NA | NA |  |  |
|  | Distal colon cancer | IVW | 0.93 (0.83 , 1.05) | 0.25 | NA | NA | NA | NA |  |  |
|  | Distal colon cancer | Egger | 0.86 (0.71 , 1.03) | 0.10 | NA | NA | NA | NA |  |  |
| **Men - All genome-wide pQTLs as IVs** | | | | | | | | |  |  |
|  | CRC | Inverse variance weighted | 1.05 (0.97 , 1.13) | 0.25 | NA | NA | NA | NA |  |  |
|  | CRC | MR Egger | 1.00 (0.88 , 1.15) | 0.97 | NA | NA | NA | NA |  |  |
|  | CRC | Simple median | 1.06 (0.94 , 1.20) | 0.32 | NA | NA | NA | NA |  |  |
|  | CRC | Weighted median | 1.01 (0.91 , 1.12) | 0.85 | NA | NA | NA | NA |  |  |
|  | CRC | Weighted mode | 1.03 (0.93 , 1.14) | 0.62 | NA | NA | NA | NA |  |  |
|  | Colon | Inverse variance weighted | 0.99 (0.88 , 1.10) | 0.81 | NA | NA | NA | NA |  |  |
|  | Colon | MR Egger | 0.90 (0.73 , 1.11) | 0.33 | NA | NA | NA | NA |  |  |
|  | Colon | Simple median | 1.04 (0.88 , 1.21) | 0.66 | NA | NA | NA | NA |  |  |
|  | Colon | Weighted median | 0.97 (0.84 , 1.13) | 0.73 | NA | NA | NA | NA |  |  |
|  | Colon | Weighted mode | 1.02 (0.81 , 1.28) | 0.86 | NA | NA | NA | NA |  |  |
|  | Rectal | Inverse variance weighted | 0.97 (0.85 , 1.10) | 0.59 | NA | NA | NA | NA |  |  |
|  | Rectal | MR Egger | 0.81 (0.65 , 1.01) | 0.09 | NA | NA | NA | NA |  |  |
|  | Rectal | Simple median | 1.12 (0.92 , 1.37) | 0.26 | NA | NA | NA | NA |  |  |
|  | Rectal | Weighted median | 0.91 (0.77 , 1.07) | 0.24 | NA | NA | NA | NA |  |  |
|  | Rectal | Weighted mode | 0.87 (0.72 , 1.06) | 0.19 | NA | NA | NA | NA |  |  |
|  | Proximal colon cancer | Inverse variance weighted | 0.96 (0.83 , 1.11) | 0.57 | NA | NA | NA | NA |  |  |
|  | Proximal colon cancer | MR Egger | 0.96 (0.72 , 1.28) | 0.78 | NA | NA | NA | NA |  |  |
|  | Proximal colon cancer | Simple median | 0.83 (0.67 , 1.03) | 0.10 | NA | NA | NA | NA |  |  |
|  | Proximal colon cancer | Weighted median | 0.83 (0.69 , 1.00) | 0.05 | NA | NA | NA | NA |  |  |
|  | Proximal colon cancer | Weighted mode | 0.79 (0.55 , 1.13) | 0.22 | NA | NA | NA | NA |  |  |
|  | Distal colon cancer | Inverse variance weighted | 1.03 (0.90 , 1.18) | 0.68 | NA | NA | NA | NA |  |  |
|  | Distal colon cancer | MR Egger | 0.91 (0.71 , 1.16) | 0.47 | NA | NA | NA | NA |  |  |
|  | Distal colon cancer | Simple median | 1.09 (0.90 , 1.33) | 0.38 | NA | NA | NA | NA |  |  |
|  | Distal colon cancer | Weighted median | 1.01 (0.85 , 1.21) | 0.89 | NA | NA | NA | NA |  |  |
|  | Distal colon cancer | Weighted mode | 1.00 (0.82 , 1.21) | 0.99 | NA | NA | NA | NA |  |  |
| **Men – cis- pQTLs as IVs** | | | | | | | | |  |  |
|  | CRC | Inverse variance weighted | 1.01 (0.91 , 1.11) | 0.88 | NA | NA | NA | NA |  |  |
|  | CRC | MR Egger | 1.03 (0.86 , 1.23) | 0.79 | NA | NA | NA | NA |  |  |
|  | CRC | Simple median | 1.03 (0.92 , 1.16) | 0.63 | NA | NA | NA | NA |  |  |
|  | CRC | Weighted median | 1.03 (0.93 , 1.15) | 0.58 | NA | NA | NA | NA |  |  |
|  | CRC | Weighted mode | 1.02 (0.91 , 1.14) | 0.79 | NA | NA | NA | NA |  |  |
|  | Colon | Inverse variance weighted | 0.97 (0.82 , 1.14) | 0.69 | NA | NA | NA | NA |  |  |
|  | Colon | MR Egger | 0.89 (0.65 , 1.24) | 0.57 | NA | NA | NA | NA |  |  |
|  | Colon | Simple median | 0.94 (0.80 , 1.11) | 0.49 | NA | NA | NA | NA |  |  |
|  | Colon | Weighted median | 0.99 (0.83 , 1.17) | 0.88 | NA | NA | NA | NA |  |  |
|  | Colon | Weighted mode | 0.98 (0.78 , 1.25) | 0.90 | NA | NA | NA | NA |  |  |
|  | Rectal | Inverse variance weighted | 0.84 (0.72 , 0.99) | 0.03 | NA | NA | NA | NA |  |  |
|  | Rectal | MR Egger | 0.83 (0.63 , 1.08) | 0.30 | NA | NA | NA | NA |  |  |
|  | Rectal | Simple median | 0.84 (0.70 , 1.02) | 0.08 | NA | NA | NA | NA |  |  |
|  | Rectal | Weighted median | 0.87 (0.72 , 1.04) | 0.12 | NA | NA | NA | NA |  |  |
|  | Rectal | Weighted mode | 0.87 (0.70 , 1.08) | 0.30 | NA | NA | NA | NA |  |  |
|  | Proximal colon cancer | Inverse variance weighted | 1.03 (0.78 , 1.38) | 0.83 | NA | NA | NA | NA |  |  |
|  | Proximal colon cancer | MR Egger | 0.83 (0.51 , 1.34) | 0.52 | NA | NA | NA | NA |  |  |
|  | Proximal colon cancer | Simple median | 0.98 (0.75 , 1.27) | 0.87 | NA | NA | NA | NA |  |  |
|  | Proximal colon cancer | Weighted median | 0.97 (0.75 , 1.25) | 0.81 | NA | NA | NA | NA |  |  |
|  | Proximal colon cancer | Weighted mode | 0.83 (0.49 , 1.40) | 0.53 | NA | NA | NA | NA |  |  |
|  | Distal colon cancer | Inverse variance weighted | 0.95 (0.80 , 1.12) | 0.51 | NA | NA | NA | NA |  |  |
|  | Distal colon cancer | MR Egger | 1.05 (0.80 , 1.39) | 0.75 | NA | NA | NA | NA |  |  |
|  | Distal colon cancer | Simple median | 0.94 (0.77 , 1.15) | 0.54 | NA | NA | NA | NA |  |  |
|  | Distal colon cancer | Weighted median | 1.00 (0.82 , 1.22) | 0.98 | NA | NA | NA | NA |  |  |
|  | Distal colon cancer | Weighted mode | 1.00 (0.80 , 1.24) | 0.99 | NA | NA | NA | NA |  |  |
| **Men - “The partially independent cis-pQTLs” as IVs *** | | | | | | | | |  |  |
|  | CRC | IVW | 0.99 (0.90 , 1.10) | 0.89 | NA | NA | NA | NA |  |  |
|  | CRC | Egger | 0.90 (0.77 , 1.05) | 0.18 | NA | NA | NA | NA |  |  |
|  | Colon cancer | IVW | 0.99 (0.90 , 1.10) | 0.89 | NA | NA | NA | NA |  |  |
|  | Colon cancer | Egger | 0.90 (0.77 , 1.05) | 0.18 | NA | NA | NA | NA |  |  |
|  | Rectal cancer | IVW | 0.99 (0.90 , 1.10) | 0.89 | NA | NA | NA | NA |  |  |
|  | Rectal cancer | Egger | 0.90 (0.77 , 1.05) | 0.18 | NA | NA | NA | NA |  |  |
|  | Proximal colon cancer | IVW | 0.99 (0.90 , 1.10) | 0.89 | NA | NA | NA | NA |  |  |
|  | Proximal colon cancer | Egger | 0.90 (0.77 , 1.05) | 0.18 | NA | NA | NA | NA |  |  |
|  | Distal colon cancer | IVW | 0.99 (0.90 , 1.10) | 0.89 | NA | NA | NA | NA |  |  |
|  | Distal colon cancer | Egger | 0.90 (0.77 , 1.05) | 0.18 | NA | NA | NA | NA |  |  |
| **Women - All genome-wide pQTLs as IVs** | | | | | | | | |  |  |
|  | CRC | Inverse variance weighted | 0.98 (0.91 , 1.06) | 0.59 | NA | NA | NA | NA |  |  |
|  | CRC | MR Egger | 0.92 (0.81 , 1.04) | 0.21 | NA | NA | NA | NA |  |  |
|  | CRC | Simple median | 1.03 (0.91 , 1.16) | 0.64 | NA | NA | NA | NA |  |  |
|  | CRC | Weighted median | 0.93 (0.84 , 1.03) | 0.18 | NA | NA | NA | NA |  |  |
|  | CRC | Weighted mode | 0.94 (0.85 , 1.04) | 0.27 | NA | NA | NA | NA |  |  |
|  | Colon | Inverse variance weighted | 0.97 (0.88 , 1.07) | 0.51 | NA | NA | NA | NA |  |  |
|  | Colon | MR Egger | 0.88 (0.74 , 1.04) | 0.17 | NA | NA | NA | NA |  |  |
|  | Colon | Simple median | 1.06 (0.90 , 1.24) | 0.51 | NA | NA | NA | NA |  |  |
|  | Colon | Weighted median | 0.90 (0.79 , 1.04) | 0.15 | NA | NA | NA | NA |  |  |
|  | Colon | Weighted mode | 0.91 (0.78 , 1.05) | 0.21 | NA | NA | NA | NA |  |  |
|  | Rectal | Inverse variance weighted | 1.05 (0.92 , 1.20) | 0.47 | NA | NA | NA | NA |  |  |
|  | Rectal | MR Egger | 0.98 (0.78 , 1.24) | 0.88 | NA | NA | NA | NA |  |  |
|  | Rectal | Simple median | 1.01 (0.83 , 1.23) | 0.90 | NA | NA | NA | NA |  |  |
|  | Rectal | Weighted median | 1.01 (0.85 , 1.21) | 0.87 | NA | NA | NA | NA |  |  |
|  | Rectal | Weighted mode | 1.01 (0.83 , 1.23) | 0.94 | NA | NA | NA | NA |  |  |
|  | Proximal colon cancer | Inverse variance weighted | 0.90 (0.80 , 1.02) | 0.10 | NA | NA | NA | NA |  |  |
|  | Proximal colon cancer | MR Egger | 0.84 (0.68 , 1.05) | 0.16 | NA | NA | NA | NA |  |  |
|  | Proximal colon cancer | Simple median | 0.87 (0.73 , 1.04) | 0.12 | NA | NA | NA | NA |  |  |
|  | Proximal colon cancer | Weighted median | 0.87 (0.74 , 1.03) | 0.10 | NA | NA | NA | NA |  |  |
|  | Proximal colon cancer | Weighted mode | 0.88 (0.74 , 1.05) | 0.18 | NA | NA | NA | NA |  |  |
|  | Distal colon cancer | Inverse variance weighted | 1.02 (0.87 , 1.19) | 0.82 | NA | NA | NA | NA |  |  |
|  | Distal colon cancer | MR Egger | 0.92 (0.68 , 1.23) | 0.57 | NA | NA | NA | NA |  |  |
|  | Distal colon cancer | Simple median | 1.11 (0.89 , 1.39) | 0.33 | NA | NA | NA | NA |  |  |
|  | Distal colon cancer | Weighted median | 0.91 (0.75 , 1.10) | 0.33 | NA | NA | NA | NA |  |  |
|  | Distal colon cancer | Weighted mode | 0.86 (0.68 , 1.10) | 0.26 | NA | NA | NA | NA |  |  |
| **Women - cis pQTLs as IVs** | | | | | | | | |  |  |
|  | CRC | Inverse variance weighted | 0.94 (0.85 , 1.04) | 0.21 | NA | NA | NA | NA |  |  |
|  | CRC | MR Egger | 0.91 (0.79 , 1.06) | 0.35 | NA | NA | NA | NA |  |  |
|  | CRC | Simple median | 0.94 (0.84 , 1.06) | 0.33 | NA | NA | NA | NA |  |  |
|  | CRC | Weighted median | 0.93 (0.83 , 1.04) | 0.20 | NA | NA | NA | NA |  |  |
|  | CRC | Weighted mode | 0.93 (0.83 , 1.05) | 0.31 | NA | NA | NA | NA |  |  |
|  | Colon | Inverse variance weighted | 0.90 (0.80 , 1.02) | 0.11 | NA | NA | NA | NA |  |  |
|  | Colon | MR Egger | 0.86 (0.71 , 1.06) | 0.29 | NA | NA | NA | NA |  |  |
|  | Colon | Simple median | 0.89 (0.76 , 1.05) | 0.17 | NA | NA | NA | NA |  |  |
|  | Colon | Weighted median | 0.88 (0.76 , 1.01) | 0.06 | NA | NA | NA | NA |  |  |
|  | Colon | Weighted mode | 0.87 (0.74 , 1.02) | 0.19 | NA | NA | NA | NA |  |  |
|  | Rectal | Inverse variance weighted | 1.04 (0.81 , 1.35) | 0.73 | NA | NA | NA | NA |  |  |
|  | Rectal | MR Egger | 0.96 (0.59 , 1.54) | 0.87 | NA | NA | NA | NA |  |  |
|  | Rectal | Simple median | 1.04 (0.85 , 1.26) | 0.72 | NA | NA | NA | NA |  |  |
|  | Rectal | Weighted median | 1.02 (0.84 , 1.25) | 0.82 | NA | NA | NA | NA |  |  |
|  | Rectal | Weighted mode | 1.02 (0.84 , 1.24) | 0.87 | NA | NA | NA | NA |  |  |
|  | Proximal colon cancer | Inverse variance weighted | 0.87 (0.74 , 1.02) | 0.09 | NA | NA | NA | NA |  |  |
|  | Proximal colon cancer | MR Egger | 0.89 (0.68 , 1.16) | 0.47 | NA | NA | NA | NA |  |  |
|  | Proximal colon cancer | Simple median | 0.86 (0.72 , 1.04) | 0.12 | NA | NA | NA | NA |  |  |
|  | Proximal colon cancer | Weighted median | 0.87 (0.72 , 1.04) | 0.13 | NA | NA | NA | NA |  |  |
|  | Proximal colon cancer | Weighted mode | 0.87 (0.71 , 1.07) | 0.27 | NA | NA | NA | NA |  |  |
|  | Distal colon cancer | Inverse variance weighted | 0.92 (0.77 , 1.10) | 0.37 | NA | NA | NA | NA |  |  |
|  | Distal colon cancer | MR Egger | 0.79 (0.59 , 1.06) | 0.26 | NA | NA | NA | NA |  |  |
|  | Distal colon cancer | Simple median | 0.97 (0.77 , 1.21) | 0.79 | NA | NA | NA | NA |  |  |
|  | Distal colon cancer | Weighted median | 0.84 (0.69 , 1.04) | 0.11 | NA | NA | NA | NA |  |  |
|  | Distal colon cancer | Weighted mode | 0.85 (0.69 , 1.06) | 0.25 | NA | NA | NA | NA |  |  |
| **Women – “The partially independent cis-pQTLs” as IVs *** | | | | | | | | |  |  |
|  | CRC | IVW | 0.99 (0.90 , 1.10) | 0.89 | NA | NA | NA | NA |  |  |
|  | CRC | Egger | 0.90 (0.77 , 1.05) | 0.18 | NA | NA | NA | NA |  |  |
|  | Colon cancer | IVW | 0.99 (0.90 , 1.10) | 0.89 | NA | NA | NA | NA |  |  |
|  | Colon cancer | Egger | 0.90 (0.77 , 1.05) | 0.18 | NA | NA | NA | NA |  |  |
|  | Rectal cancer | IVW | 0.99 (0.90 , 1.10) | 0.89 | NA | NA | NA | NA |  |  |
|  | Rectal cancer | Egger | 0.90 (0.77 , 1.05) | 0.18 | NA | NA | NA | NA |  |  |
|  | Proximal colon cancer | IVW | 0.99 (0.90 , 1.10) | 0.89 | NA | NA | NA | NA |  |  |
|  | Proximal colon cancer | Egger | 0.90 (0.77 , 1.05) | 0.18 | NA | NA | NA | NA |  |  |
|  | Distal colon cancer | IVW | 0.99 (0.90 , 1.10) | 0.89 | NA | NA | NA | NA |  |  |
|  | Distal colon cancer | Egger | 0.90 (0.77 , 1.05) | 0.18 | NA | NA | NA | NA |  |  |

ORs (95%C) estimated for the effect of one SD increase in genetically predicted serum resistin concentrations and risk of CRC. The number of controls and CRC subtype cases in GECCO were as follows: 67347 controls, 58131 CRC cases, 31083 colon cases, 13857 proximal cases, 15306 distal cases, and 15775 rectal cases; and in FinnGen were: 4957 CRC, 2989 colon cancer, and 1832 rectal cancer, and 245 442 controls.

Cis pQTLs including variants residing within 1 megabase upstream or downstream of the transcription start site of the resistin-encoded gene (RETN gene) and tran pQTLs including variants residing elsewhere in the genome. The partially independent cis-pQTLs including variants residing within ±100kb upstream or downstream of the transcription start site of the RETN gene that had low linkage disequilibrium LDs (r2 <= 0.1) and were at a distance ranging from 10kb to 100 bases apart from each other. Here, 8 variants (rs10401670, rs2161490, rs34124816, rs35547567, rs3745367, rs4804766, rs62110711, rs77509849 were considered in the set of “partially independent cis-pQTLs”. The MR analysis accounted for the correlation matrix between these “partially independent cis-pQTLs” variants.

Furthermore, robust methods in MR analysis were applied with descriptions as follows:

- **Inverse variance weighted (IVW)** models are assumed that the pleiotropic effect is independent of the instrument strength (InSIDE assumption) and the mean pleiotropic effect is zero (balanced pleiotropy) and therefore sensitive to outliers (pleiotropy often manifests as the presence of genetic variants with outlying ratio estimates).
- **MR-Egger** relaxes the assumption that the average pleiotropic effect is zero (to allow for ‘directional pleiotropy’) by introducing an intercept in the regression model, however, MR-Egger often suffers from low power.
- **Simple and Weighted median models** are more robust to outliers. If the majority of variants (i.e. >50%) are valid instruments, the median will be an accurate estimate of the true causal effect, especially with large sample sizes.
- **Weighted mode models**: take ratio estimates, fit a smooth curve (Kernel) to model their distribution, and use the mode of that distribution as the overall causal effect estimate. Like the median, the mode models are more robust to outliers than IVW. Mode-based models only require a plurality of genetic variants to be valid and could be sensitive to weak instruments.

Supplementary Table 2: Leave-one instrumental variable out and effect estimates of the relationship between genetically determined circulating resistin concentrations and risk of colorectal cancer using Mendelian Randomization with inverse variance-weighted method.

| SNPs | **OR (95%CI) *** | p-value |
| --- | --- | --- |
| All SNPs | 1.01 (0.96 , 1.06) | 0.67 |
| All SNPs excluding rs10103048 | 1.01 (0.96 , 1.07) | 0.69 |
| All SNPs excluding rs10401670 | 1.02 (0.96 , 1.08) | 0.54 |
| All SNPs excluding rs17405635 | 1.01 (0.96 , 1.07) | 0.63 |
| All SNPs excluding rs2239619 | 1.00 (0.95 , 1.06) | 0.91 |
| All SNPs excluding rs3087852 | 1.01 (0.96 , 1.06) | 0.77 |
| All SNPs excluding rs34861192 | 1.03 (0.97 , 1.10) | 0.29 |
| All SNPs excluding rs3745367 | 1.01 (0.96 , 1.07) | 0.75 |
| All SNPs excluding rs4134826 | 1.01 (0.96 , 1.07) | 0.62 |
| All SNPs excluding rs445 | 1.01 (0.96 , 1.07) | 0.60 |
| All SNPs excluding rs6775731 | 1.01 (0.96 , 1.06) | 0.78 |
| All SNPs excluding rs73008259 | 1.01 (0.95 , 1.06) | 0.85 |
| All SNPs excluding rs7589428 | 1.01 (0.96 , 1.07) | 0.61 |
| All SNPs excluding rs77691416 | 1.00 (0.95 , 1.06) | 0.87 |
| All cis-SNPs | 0.98 (0.92 , 1.04) | 0.49 |
| All cis SNPs excluding rs10401670 | 0.99 (0.92 , 1.06) | 0.75 |
| All cis SNPs excluding rs34861192 | 0.99 (0.90 , 1.09) | 0.82 |
| All cis SNPs excluding rs3745367 | 0.97 (0.90 , 1.04) | 0.36 |
| All cis SNPs excluding rs4134826 | 0.98 (0.92 , 1.05) | 0.53 |
| All trans SNPs | 1.07 (0.98 , 1.17) | 0.11 |
| All trans SNPs excluding rs10103048 | 1.08 (0.98 , 1.18) | 0.11 |
| All trans SNPs excluding rs17405635 | 1.09 (0.99 , 1.19) | 0.07 |
| All trans SNPs excluding rs2239619 | 1.05 (0.96 , 1.14) | 0.29 |
| All trans SNPs excluding rs3087852 | 1.08 (0.98 , 1.18) | 0.12 |
| All trans SNPs excluding rs445 | 1.09 (0.99 , 1.19) | 0.07 |
| All trans SNPs excluding rs6775731 | 1.07 (0.98 , 1.17) | 0.15 |
| All trans SNPs excluding rs73008259 | 1.06 (0.97 , 1.16) | 0.18 |
| All trans SNPs excluding rs7589428 | 1.08 (0.99 , 1.18) | 0.09 |
| All trans SNPs excluding rs77691416 | 1.06 (0.97 , 1.16) | 0.20 |

*Pooled estimates were derived from meta-analyses by inverse variance-weighted random effects models combining estimates of the association between genetically determined resistin levels resulting from SCALLOP-GECCO and SCALLOP-FinnGen.

Supplementary Table 3: Traits of each resistin-pQTL (in addition to resistin concentrations)

| No. | SNP | Chr | Position | Gene closest | Gene-cis | Effect allele | Reference allele | Traits ^a^ |
| --- | --- | --- | --- | --- | --- | --- | --- | --- |
| 1 | rs17405635 | 2 | 43355763 | ZFP36L2 | trans | A | G | Percentage or count of monocyte, neutrophil, granulocyte |
| 2 | rs7589428 | 2 | 43561771 | THADA | trans | A | G | Percentage or count of lymphocyte, neutrophil, eosinophil |
| 3 | rs6775731 | 3 | 128306894 | RPN1 | trans | T | C | Percentage or count of monocyte, neutrophil, basophil, eosinophil, granulocyte, myeloid white cell |
| 4 | rs2239619 | 6 | 52453220 | TRAM2 | trans | A | C | **LDL or total cholesterol** |
| 5 | rs73008259 | 6 | 52450548 | TRAM2 | trans | A | T | Percentage or count of monocyte, neutrophil, basophil, eosinophil, granulocyte |
| 6 | rs77691416 | 6 | 144354119 | PLAGL1 | trans | A | C | Percentage or count of monocyte, granulocyte |
| 7 | rs445 | 7 | 92408370 | CDK6 | trans | T | C | Percentage or count of  neutrophil, basophil, eosinophil, granulocyte, myeloid white cell, white blood cell |
| 8 | rs10103048 | 8 | 130602281 | GSDMC | trans | A | C | Percentage or count of lymphocyte, monocyte, neutrophil, basophil, eosinophil, granulocyte, myeloid white cell, white blood cell |
| 9 | rs3087852 | 17 | 38137364 | PSMD3 | trans | A | G | Percentage or count of lymphocyte, monocyte, neutrophil, basophil, eosinophil, granulocyte, myeloid white cell, white blood cell |
| 10 | rs4134826 | 19 | 7733575 | RETN | **RETN** | A | G | No data available |
| 11 | rs3745367 | 19 | 7692076 | XAB2 | **RETN** | T | C | Circulating resistin levels |
| 12 | rs34861192 | 19 | 7734511 | RETN | **RETN** | A | G | Treatment with sodium thyroxine |
| 13 | rs10401670 | 19 | 7742802 | MCEMP1 | **RETN** | T | C | No data available |

1. Traits were checked and obtained from pQTLs from GWAS Catalog using phenoscanner:: phenoscanner() function in R

Supplementary Fig. 3: Funnel plot of MR estimates of the exposure-outcome against their precision for each exposure-outcome dataset.

| 1. SCALLOP & GECCO   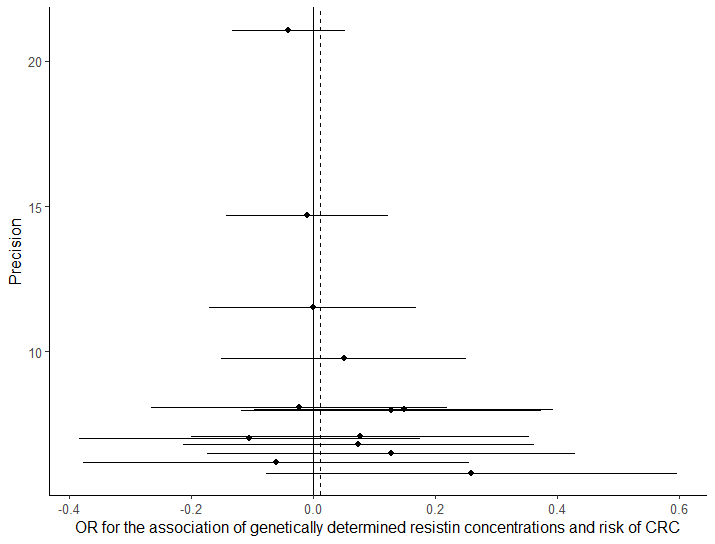 | 1. SCALLOP & FinnGen   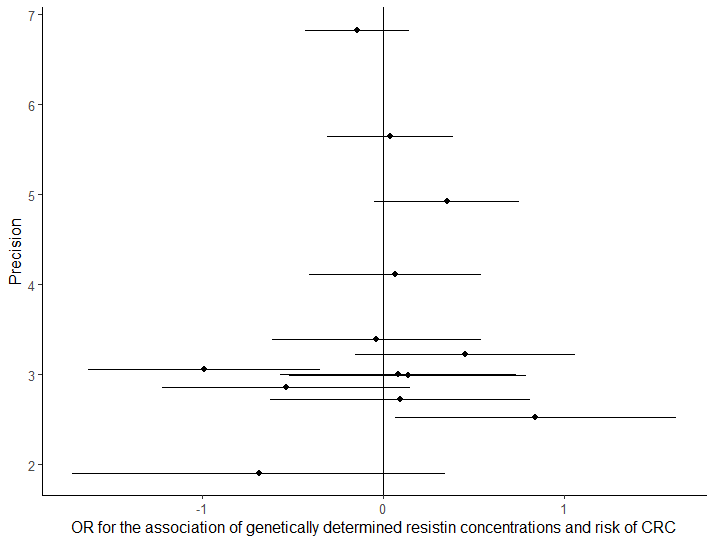 |
| --- | --- |

In the funnel plot, a genetic variant is represented by each data point. The x-axis shows the coefficient of resistin-CRC risk association was estimated for each genetic variant by the Wald ratios method (beta(CRC~variant)/ beta(resistin~variant)). The y-axis shows the precision of the corresponding estimate. The asymmetry of funnel plots is pertaining to some genetic variants strongly associated with the outcome given their low precision and could be an indication of unbalanced or directional pleiotropy.

Supplementary Table 4: Genetic summary data for CRC

| **GECCO/CCFR/ CORECT data (referred to as GECCO)** | | | | | | | | |
| --- | --- | --- | --- | --- | --- | --- | --- | --- |
| No | Outcome | SNP | Effect allele | Alternative allele | Effect allele frequency | beta | Standard error of beta | pval |
| 1 | All Colorectal Cancer | rs17405635 | A | G | 0.2623 | -0.0018 | 0.0099 | 0.8594 |
| 2 | All Colorectal Cancer | rs7589428 | A | G | 0.4931 | 0.0049 | 0.0089 | 0.5817 |
| 3 | All Colorectal Cancer | rs6775731 | T | C | 0.2828 | -0.0081 | 0.0097 | 0.4032 |
| 4 | All Colorectal Cancer | rs7746716 | A | T | 0.5419 | -0.0147 | 0.0088 | 0.09436 |
| 5 | All Colorectal Cancer | rs2239619 | A | C | 0.634 | -0.0138 | 0.0091 | 0.131 |
| 6 | All Colorectal Cancer | rs77691416 | A | C | 0.904 | 0.0179 | 0.015 | 0.233 |
| 7 | All Colorectal Cancer | rs73008259 | A | G | 0.0433 | 0.0244 | 0.0238 | 0.3055 |
| 8 | All Colorectal Cancer | rs445 | T | C | 0.1244 | 0.0051 | 0.0137 | 0.7109 |
| 9 | All Colorectal Cancer | rs10103048 | A | C | 0.4182 | 0.0045 | 0.0088 | 0.6128 |
| 10 | All Colorectal Cancer | rs3087852 | A | G | 0.4675 | -0.0044 | 0.0088 | 0.6183 |
| 11 | All Colorectal Cancer | rs4134826 | T | C | 0.8632 | 0.0092 | 0.0127 | 0.4686 |
| 12 | All Colorectal Cancer | rs34861192 | A | G | 0.0122 | -0.0436 | 0.0522 | 0.4042 |
| 13 | All Colorectal Cancer | rs3745367 | A | G | 0.2538 | 0 | 0.0104 | 0.9974 |
| 14 | All Colorectal Cancer | rs10401670 | T | C | 0.432 | -0.0014 | 0.0102 | 0.8906 |
| 15 | All Colon | rs17405635 | A | G | 0.2622 | -9.00E-04 | 0.012 | 0.9377 |
| 16 | All Colon | rs7589428 | A | G | 0.4926 | -8.00E-04 | 0.0107 | 0.9392 |
| 17 | All Colon | rs6775731 | T | C | 0.283 | -2.00E-04 | 0.0117 | 0.9877 |
| 18 | All Colon | rs7746716 | A | T | 0.5419 | -0.017 | 0.0106 | 0.1081 |
| 19 | All Colon | rs2239619 | A | C | 0.634 | -0.0171 | 0.011 | 0.121 |
| 20 | All Colon | rs77691416 | A | C | 0.9036 | 0.0252 | 0.0178 | 0.1583 |
| 21 | All Colon | rs73008259 | A | G | 0.0436 | 0.0061 | 0.0287 | 0.8318 |
| 22 | All Colon | rs445 | T | C | 0.1243 | 0.0329 | 0.0169 | 0.05193 |
| 23 | All Colon | rs10103048 | A | C | 0.418 | 0.0088 | 0.0107 | 0.4098 |
| 24 | All Colon | rs3087852 | A | G | 0.4672 | 5.00E-04 | 0.0106 | 0.9617 |
| 25 | All Colon | rs4134826 | T | C | 0.8617 | 0.0193 | 0.0152 | 0.2035 |
| 26 | All Colon | rs34861192 | A | G | 0.0118 | -0.1544 | 0.0744 | 0.03799 |
| 27 | All Colon | rs3745367 | A | G | 0.2542 | 0.0048 | 0.0127 | 0.7069 |
| 28 | All Colon | rs10401670 | T | C | 0.4319 | -0.001 | 0.0123 | 0.9384 |
| 29 | All Rectal | rs17405635 | A | G | 0.2625 | -0.0098 | 0.0156 | 0.5289 |
| 30 | All Rectal | rs7589428 | A | G | 0.493 | 0.0022 | 0.0138 | 0.876 |
| 31 | All Rectal | rs6775731 | T | C | 0.2816 | -0.0256 | 0.0151 | 0.09136 |
| 32 | All Rectal | rs7746716 | A | T | 0.5435 | -0.0129 | 0.0137 | 0.3457 |
| 33 | All Rectal | rs2239619 | A | C | 0.6354 | -0.0094 | 0.0143 | 0.5123 |
| 34 | All Rectal | rs77691416 | A | C | 0.9036 | 0.0115 | 0.0231 | 0.6183 |
| 35 | All Rectal | rs73008259 | A | G | 0.0426 | 0.017 | 0.0375 | 0.6511 |
| 36 | All Rectal | rs445 | T | C | 0.1261 | -0.0194 | 0.0224 | 0.3848 |
| 37 | All Rectal | rs10103048 | A | C | 0.4194 | -0.0056 | 0.0139 | 0.688 |
| 38 | All Rectal | rs3087852 | A | G | 0.4676 | -0.0202 | 0.0137 | 0.14 |
| 39 | All Rectal | rs4134826 | T | C | 0.8624 | -0.0074 | 0.0195 | 0.7058 |
| 40 | All Rectal | rs34861192 | A | G | 0.0126 | -0.0708 | 0.0913 | 0.4379 |
| 41 | All Rectal | rs3745367 | A | G | 0.2556 | -0.0199 | 0.0165 | 0.2256 |
| 42 | All Rectal | rs10401670 | T | C | 0.4315 | -4.00E-04 | 0.016 | 0.9817 |
| 43 | All Proximal | rs17405635 | A | G | 0.2626 | -0.0175 | 0.0155 | 0.2606 |
| 44 | All Proximal | rs7589428 | A | G | 0.4928 | -0.017 | 0.0138 | 0.2196 |
| 45 | All Proximal | rs6775731 | T | C | 0.2822 | 0.0099 | 0.0151 | 0.5149 |
| 46 | All Proximal | rs7746716 | A | T | 0.5432 | -0.0184 | 0.0137 | 0.18 |
| 47 | All Proximal | rs2239619 | A | C | 0.6355 | -0.0271 | 0.0143 | 0.0575 |
| 48 | All Proximal | rs77691416 | A | C | 0.9035 | 0.013 | 0.023 | 0.5719 |
| 49 | All Proximal | rs73008259 | A | G | 0.0433 | -0.0208 | 0.038 | 0.5839 |
| 50 | All Proximal | rs445 | T | C | 0.126 | 0.0398 | 0.0219 | 0.0695 |
| 51 | All Proximal | rs10103048 | A | C | 0.4185 | 7.00E-04 | 0.0138 | 0.9595 |
| 52 | All Proximal | rs3087852 | A | G | 0.4681 | 0.0135 | 0.0137 | 0.3249 |
| 53 | All Proximal | rs4134826 | T | C | 0.8619 | 0.028 | 0.0197 | 0.1543 |
| 54 | All Proximal | rs34861192 | A | G | 0.0121 | -0.1896 | 0.0972 | 0.0511 |
| 55 | All Proximal | rs3745367 | A | G | 0.2549 | 0.0085 | 0.0164 | 0.6054 |
| 56 | All Proximal | rs10401670 | T | C | 0.4316 | 0.0125 | 0.016 | 0.4342 |
| 57 | All Distal | rs17405635 | A | G | 0.263 | 0.0236 | 0.0157 | 0.1323 |
| 58 | All Distal | rs7589428 | A | G | 0.4933 | 0.0119 | 0.014 | 0.3962 |
| 59 | All Distal | rs6775731 | T | C | 0.2819 | -0.0034 | 0.0154 | 0.8267 |
| 60 | All Distal | rs7746716 | A | T | 0.5433 | -0.0133 | 0.014 | 0.3403 |
| 61 | All Distal | rs2239619 | A | C | 0.6356 | -0.005 | 0.0145 | 0.7316 |
| 62 | All Distal | rs77691416 | A | C | 0.9036 | 0.0269 | 0.0235 | 0.253 |
| 63 | All Distal | rs73008259 | A | G | 0.0432 | 0.0379 | 0.0378 | 0.3159 |
| 64 | All Distal | rs445 | T | C | 0.1263 | 0.0284 | 0.0222 | 0.2001 |
| 65 | All Distal | rs10103048 | A | C | 0.4196 | 0.0159 | 0.0141 | 0.2582 |
| 66 | All Distal | rs3087852 | A | G | 0.4685 | -0.005 | 0.0139 | 0.7207 |
| 67 | All Distal | rs4134826 | T | C | 0.8619 | 0.0076 | 0.02 | 0.7014 |
| 68 | All Distal | rs34861192 | A | G | 0.0123 | -0.0425 | 0.0959 | 0.6577 |
| 69 | All Distal | rs3745367 | A | G | 0.2552 | 0.0037 | 0.0167 | 0.8269 |
| 70 | All Distal | rs10401670 | T | C | 0.4311 | -0.0221 | 0.0163 | 0.1758 |
| 71 | Men Colorectal Cancer | rs17405635 | A | G | 0.2617 | -0.0072 | 0.0138 | 0.6039 |
| 72 | Men Colorectal Cancer | rs7589428 | A | G | 0.4929 | 6.00E-04 | 0.0124 | 0.9599 |
| 73 | Men Colorectal Cancer | rs6775731 | T | C | 0.2825 | -0.0186 | 0.0136 | 0.172 |
| 74 | Men Colorectal Cancer | rs7746716 | A | T | 0.5421 | -0.0239 | 0.0122 | 0.05064 |
| 75 | Men Colorectal Cancer | rs2239619 | A | C | 0.6339 | -0.0254 | 0.0128 | 0.0465 |
| 76 | Men Colorectal Cancer | rs77691416 | A | C | 0.9044 | 0.0261 | 0.021 | 0.214 |
| 77 | Men Colorectal Cancer | rs73008259 | A | G | 0.0438 | 0.044 | 0.0332 | 0.1858 |
| 78 | Men Colorectal Cancer | rs445 | T | C | 0.124 | -0.0199 | 0.0191 | 0.2985 |
| 79 | Men Colorectal Cancer | rs10103048 | A | C | 0.4186 | 0.0074 | 0.0124 | 0.5497 |
| 80 | Men Colorectal Cancer | rs3087852 | A | G | 0.4672 | 0.0101 | 0.0123 | 0.4112 |
| 81 | Men Colorectal Cancer | rs4134826 | T | C | 0.8617 | 0.0271 | 0.0177 | 0.1258 |
| 82 | Men Colorectal Cancer | rs34861192 | A | G | 0.0121 | 0.0025 | 0.0758 | 0.9737 |
| 83 | Men Colorectal Cancer | rs3745367 | A | G | 0.2552 | 0.0065 | 0.0146 | 0.6547 |
| 84 | Men Colorectal Cancer | rs10401670 | T | C | 0.4333 | 0.009 | 0.0144 | 0.5286 |
| 85 | Men Colon | rs10103048 | A | C | 0.4188 | 0.0141 | 0.0153 | 0.359 |
| 86 | Men Colon | rs17405635 | A | G | 0.2615 | -0.0061 | 0.0172 | 0.7236 |
| 87 | Men Colon | rs2239619 | A | C | 0.6341 | -0.0309 | 0.0158 | 0.05051 |
| 88 | Men Colon | rs445 | T | C | 0.1282 | 0.0208 | 0.0241 | 0.3886 |
| 89 | Men Colon | rs4134826 | T | C | 0.8621 | 0.0279 | 0.0216 | 0.1976 |
| 90 | Men Colon | rs3745367 | A | G | 0.2574 | 0.0043 | 0.0181 | 0.8117 |
| 91 | Men Colon | rs34861192 | A | G | 0.0134 | -0.1648 | 0.1146 | 0.1505 |
| 92 | Men Colon | rs7589428 | A | G | 0.4935 | -0.0065 | 0.0153 | 0.6724 |
| 93 | Men Colon | rs10401670 | T | C | 0.4324 | 0.0203 | 0.0176 | 0.2486 |
| 94 | Men Colon | rs73008259 | A | G | 0.0452 | 0.0224 | 0.0413 | 0.5873 |
| 95 | Men Colon | rs6775731 | T | C | 0.2828 | -0.0146 | 0.0168 | 0.3854 |
| 96 | Men Colon | rs7746716 | A | T | 0.5421 | -0.0329 | 0.0152 | 0.03059 |
| 97 | Men Colon | rs3087852 | A | G | 0.4678 | 0.0271 | 0.0151 | 0.07299 |
| 98 | Men Colon | rs77691416 | A | C | 0.9037 | 0.0158 | 0.0255 | 0.5358 |
| 99 | Men Rectal | rs10103048 | A | C | 0.4188 | 0.0068 | 0.0185 | 0.713 |
| 100 | Men Rectal | rs17405635 | A | G | 0.2617 | -0.0152 | 0.0207 | 0.4626 |
| 101 | Men Rectal | rs2239619 | A | C | 0.6342 | -0.0203 | 0.0189 | 0.2846 |
| 102 | Men Rectal | rs445 | T | C | 0.128 | -0.05 | 0.0298 | 0.09326 |
| 103 | Men Rectal | rs4134826 | T | C | 0.8618 | 0.0383 | 0.0261 | 0.1419 |
| 104 | Men Rectal | rs3745367 | A | G | 0.2571 | -0.0141 | 0.0218 | 0.5178 |
| 105 | Men Rectal | rs34861192 | A | G | 0.0127 | -0.2418 | 0.1375 | 0.07871 |
| 106 | Men Rectal | rs7589428 | A | G | 0.4934 | -0.0102 | 0.0184 | 0.58 |
| 107 | Men Rectal | rs10401670 | T | C | 0.4311 | -0.0115 | 0.0213 | 0.5876 |
| 108 | Men Rectal | rs73008259 | A | G | 0.0446 | 0.0327 | 0.0495 | 0.5092 |
| 109 | Men Rectal | rs6775731 | T | C | 0.2825 | -0.0204 | 0.0202 | 0.3108 |
| 110 | Men Rectal | rs7746716 | A | T | 0.5425 | -0.0184 | 0.0182 | 0.3113 |
| 111 | Men Rectal | rs3087852 | A | G | 0.4665 | -0.0177 | 0.0183 | 0.3312 |
| 112 | Men Rectal | rs77691416 | A | C | 0.9035 | 0.0214 | 0.0309 | 0.4887 |
| 113 | Men Proximal | rs10103048 | A | C | 0.4195 | -0.0145 | 0.0202 | 0.4718 |
| 114 | Men Proximal | rs17405635 | A | G | 0.2618 | -0.0269 | 0.0227 | 0.2342 |
| 115 | Men Proximal | rs2239619 | A | C | 0.6371 | -0.0288 | 0.0208 | 0.1666 |
| 116 | Men Proximal | rs445 | T | C | 0.1306 | 0.0161 | 0.0319 | 0.6144 |
| 117 | Men Proximal | rs4134826 | T | C | 0.8621 | 0.0164 | 0.0285 | 0.5657 |
| 118 | Men Proximal | rs3745367 | A | G | 0.2588 | 0.0167 | 0.0239 | 0.4842 |
| 119 | Men Proximal | rs34861192 | A | G | 0.0132 | -0.2584 | 0.1579 | 0.1018 |
| 120 | Men Proximal | rs7589428 | A | G | 0.4941 | -0.0261 | 0.0202 | 0.1968 |
| 121 | Men Proximal | rs10401670 | T | C | 0.4319 | 0.0504 | 0.0235 | 0.03172 |
| 122 | Men Proximal | rs73008259 | A | G | 0.0452 | 0.0111 | 0.055 | 0.8401 |
| 123 | Men Proximal | rs6775731 | T | C | 0.2816 | 0.0031 | 0.0222 | 0.8904 |
| 124 | Men Proximal | rs7746716 | A | T | 0.5445 | -0.02 | 0.02 | 0.3189 |
| 125 | Men Proximal | rs3087852 | A | G | 0.4685 | 0.0253 | 0.02 | 0.2055 |
| 126 | Men Proximal | rs77691416 | A | C | 0.9037 | -7.00E-04 | 0.0334 | 0.9834 |
| 127 | Men Distal | rs10103048 | A | C | 0.4197 | 0.0398 | 0.0196 | 0.04195 |
| 128 | Men Distal | rs17405635 | A | G | 0.2624 | 0.0166 | 0.0218 | 0.4453 |
| 129 | Men Distal | rs2239619 | A | C | 0.6357 | -0.0343 | 0.0201 | 0.08829 |
| 130 | Men Distal | rs445 | T | C | 0.1296 | 0.0197 | 0.0306 | 0.5198 |
| 131 | Men Distal | rs4134826 | T | C | 0.8619 | 0.0339 | 0.0277 | 0.2208 |
| 132 | Men Distal | rs3745367 | A | G | 0.2574 | -5.00E-04 | 0.0232 | 0.9811 |
| 133 | Men Distal | rs34861192 | A | G | 0.0143 | 0.0259 | 0.1415 | 0.8545 |
| 134 | Men Distal | rs7589428 | A | G | 0.4943 | 0.0085 | 0.0195 | 0.6639 |
| 135 | Men Distal | rs10401670 | T | C | 0.4311 | -0.0178 | 0.0227 | 0.4334 |
| 136 | Men Distal | rs73008259 | A | G | 0.0451 | 0.0434 | 0.0527 | 0.4105 |
| 137 | Men Distal | rs6775731 | T | C | 0.2817 | -0.0245 | 0.0215 | 0.2544 |
| 138 | Men Distal | rs7746716 | A | T | 0.5431 | -0.0433 | 0.0194 | 0.02572 |
| 139 | Men Distal | rs3087852 | A | G | 0.4688 | 0.0255 | 0.0193 | 0.1853 |
| 140 | Men Distal | rs77691416 | A | C | 0.9035 | 0.0104 | 0.0325 | 0.748 |
| 141 | Women Colorectal Cancer | rs17405635 | A | G | 0.2627 | 0.0039 | 0.0142 | 0.7817 |
| 142 | Women Colorectal Cancer | rs7589428 | A | G | 0.4925 | 0.0078 | 0.0127 | 0.5409 |
| 143 | Women Colorectal Cancer | rs6775731 | T | C | 0.2836 | 0.0044 | 0.014 | 0.7523 |
| 144 | Women Colorectal Cancer | rs7746716 | A | T | 0.5421 | -0.0048 | 0.0126 | 0.7033 |
| 145 | Women Colorectal Cancer | rs2239619 | A | C | 0.6341 | -0.002 | 0.0131 | 0.8796 |
| 146 | Women Colorectal Cancer | rs77691416 | A | C | 0.9031 | 0.0097 | 0.0215 | 0.6511 |
| 147 | Women Colorectal Cancer | rs73008259 | A | G | 0.0433 | 5.00E-04 | 0.0343 | 0.9878 |
| 148 | Women Colorectal Cancer | rs445 | T | C | 0.1242 | 0.0312 | 0.0198 | 0.1157 |
| 149 | Women Colorectal Cancer | rs10103048 | A | C | 0.4175 | 0.0017 | 0.0127 | 0.894 |
| 150 | Women Colorectal Cancer | rs3087852 | A | G | 0.467 | -0.0211 | 0.0126 | 0.0935 |
| 151 | Women Colorectal Cancer | rs4134826 | T | C | 0.8621 | -0.0147 | 0.0182 | 0.4205 |
| 152 | Women Colorectal Cancer | rs34861192 | A | G | 0.0119 | -0.0779 | 0.0737 | 0.2906 |
| 153 | Women Colorectal Cancer | rs3745367 | A | G | 0.2531 | -0.0053 | 0.015 | 0.7244 |
| 154 | Women Colorectal Cancer | rs10401670 | T | C | 0.4317 | -0.0154 | 0.0147 | 0.2937 |
| 155 | Women Colon | rs10103048 | A | C | 0.4174 | 0.0051 | 0.0151 | 0.7367 |
| 156 | Women Colon | rs17405635 | A | G | 0.263 | 0.0064 | 0.0169 | 0.7029 |
| 157 | Women Colon | rs2239619 | A | C | 0.6328 | -0.0061 | 0.0156 | 0.6946 |
| 158 | Women Colon | rs445 | T | C | 0.1274 | 0.0442 | 0.0241 | 0.06625 |
| 159 | Women Colon | rs4134826 | T | C | 0.8623 | 0.0083 | 0.0215 | 0.6984 |
| 160 | Women Colon | rs3745367 | A | G | 0.2545 | 0.0065 | 0.0179 | 0.7167 |
| 161 | Women Colon | rs34861192 | A | G | 0.0123 | -0.1432 | 0.1028 | 0.1637 |
| 162 | Women Colon | rs7589428 | A | G | 0.4923 | 0.0054 | 0.0151 | 0.7178 |
| 163 | Women Colon | rs10401670 | T | C | 0.4296 | -0.0234 | 0.0174 | 0.1773 |
| 164 | Women Colon | rs73008259 | A | G | 0.0442 | -0.0023 | 0.0403 | 0.955 |
| 165 | Women Colon | rs6775731 | T | C | 0.2835 | 0.0147 | 0.0165 | 0.3721 |
| 166 | Women Colon | rs7746716 | A | T | 0.5412 | -0.0033 | 0.015 | 0.823 |
| 167 | Women Colon | rs3087852 | A | G | 0.4662 | -0.0241 | 0.015 | 0.1067 |
| 168 | Women Colon | rs77691416 | A | C | 0.9034 | 0.0347 | 0.0251 | 0.1681 |
| 169 | Women Rectal | rs10103048 | A | C | 0.4194 | -0.0205 | 0.0212 | 0.3336 |
| 170 | Women Rectal | rs17405635 | A | G | 0.2628 | -8.00E-04 | 0.0239 | 0.9716 |
| 171 | Women Rectal | rs2239619 | A | C | 0.6341 | -0.001 | 0.022 | 0.9619 |
| 172 | Women Rectal | rs445 | T | C | 0.1297 | 0.0259 | 0.0341 | 0.4471 |
| 173 | Women Rectal | rs4134826 | T | C | 0.8637 | -0.0711 | 0.0296 | 0.01621 |
| 174 | Women Rectal | rs3745367 | A | G | 0.2563 | -0.0238 | 0.0253 | 0.3456 |
| 175 | Women Rectal | rs34861192 | A | G | 0.0126 | 0.0143 | 0.1411 | 0.9192 |
| 176 | Women Rectal | rs7589428 | A | G | 0.4924 | 0.0189 | 0.0211 | 0.3699 |
| 177 | Women Rectal | rs10401670 | T | C | 0.4303 | 0.0085 | 0.0243 | 0.7275 |
| 178 | Women Rectal | rs73008259 | A | G | 0.0436 | -0.0143 | 0.0582 | 0.8065 |
| 179 | Women Rectal | rs6775731 | T | C | 0.2821 | -0.0296 | 0.0232 | 0.202 |
| 180 | Women Rectal | rs7746716 | A | T | 0.5426 | -0.0113 | 0.0211 | 0.59 |
| 181 | Women Rectal | rs3087852 | A | G | 0.4673 | -0.023 | 0.021 | 0.2731 |
| 182 | Women Rectal | rs77691416 | A | C | 0.9031 | -0.0021 | 0.0351 | 0.9523 |
| 183 | Women Proximal | rs10103048 | A | C | 0.4181 | 0.0147 | 0.0189 | 0.4361 |
| 184 | Women Proximal | rs17405635 | A | G | 0.2633 | -0.0093 | 0.0212 | 0.6622 |
| 185 | Women Proximal | rs2239619 | A | C | 0.6334 | -0.0247 | 0.0195 | 0.2042 |
| 186 | Women Proximal | rs445 | T | C | 0.1288 | 0.0582 | 0.0301 | 0.05291 |
| 187 | Women Proximal | rs4134826 | T | C | 0.8626 | 0.0368 | 0.0272 | 0.1752 |
| 188 | Women Proximal | rs3745367 | A | G | 0.2554 | 6.00E-04 | 0.0225 | 0.9772 |
| 189 | Women Proximal | rs34861192 | A | G | 0.0134 | -0.1509 | 0.1368 | 0.27 |
| 190 | Women Proximal | rs7589428 | A | G | 0.4924 | -0.0093 | 0.0189 | 0.6216 |
| 191 | Women Proximal | rs10401670 | T | C | 0.4296 | -0.0241 | 0.0219 | 0.2717 |
| 192 | Women Proximal | rs73008259 | A | G | 0.0436 | -0.0386 | 0.0516 | 0.4541 |
| 193 | Women Proximal | rs6775731 | T | C | 0.2828 | 0.0174 | 0.0206 | 0.3976 |
| 194 | Women Proximal | rs7746716 | A | T | 0.5418 | -0.0172 | 0.0187 | 0.3579 |
| 195 | Women Proximal | rs3087852 | A | G | 0.467 | 0.0019 | 0.0187 | 0.9203 |
| 196 | Women Proximal | rs77691416 | A | C | 0.9032 | 0.0261 | 0.0315 | 0.4074 |
| 197 | Women Distal | rs10103048 | A | C | 0.4195 | -0.0041 | 0.0206 | 0.8429 |
| 198 | Women Distal | rs17405635 | A | G | 0.2635 | 0.0345 | 0.023 | 0.1327 |
| 199 | Women Distal | rs2239619 | A | C | 0.6343 | 0.0266 | 0.0213 | 0.2112 |
| 200 | Women Distal | rs445 | T | C | 0.1298 | 0.0398 | 0.0327 | 0.2235 |
| 201 | Women Distal | rs4134826 | T | C | 0.8632 | -0.028 | 0.0291 | 0.3346 |
| 202 | Women Distal | rs3745367 | A | G | 0.2559 | 0.013 | 0.0244 | 0.595 |
| 203 | Women Distal | rs34861192 | A | G | 0.0136 | -0.1863 | 0.1512 | 0.2178 |
| 204 | Women Distal | rs7589428 | A | G | 0.4925 | 0.0183 | 0.0205 | 0.374 |
| 205 | Women Distal | rs10401670 | T | C | 0.4294 | -0.026 | 0.0238 | 0.2746 |
| 206 | Women Distal | rs73008259 | A | G | 0.044 | 0.0507 | 0.055 | 0.3561 |
| 207 | Women Distal | rs6775731 | T | C | 0.2827 | 0.0214 | 0.0225 | 0.3398 |
| 208 | Women Distal | rs7746716 | A | T | 0.5427 | 0.0193 | 0.0204 | 0.3439 |
| 209 | Women Distal | rs3087852 | A | G | 0.4676 | -0.0379 | 0.0204 | 0.06356 |
| 210 | Women Distal | rs77691416 | A | C | 0.9035 | 0.0475 | 0.0345 | 0.1685 |
| **FinnGen** | | | | | | | | |
| No | Outcome | SNP | Effect allele | Alternative allele | Effect allele frequency | beta | Standard error of beta | pval |
| 1 | All Colorectal Cancer | rs17405635 | A | G | 0.258291 | -0.00306 | 0.023568 | 0.896546 |
| 2 | All Colorectal Cancer | rs7589428 | G | A | 0.490411 | 0.062378 | 0.020645 | 0.002516 |
| 3 | All Colorectal Cancer | rs6775731 | C | T | 0.722923 | 0.005839 | 0.023145 | 0.800837 |
| 4 | All Colorectal Cancer | rs7746716 | T | A | 0.518509 | 0.04096 | 0.020788 | 0.048799 |
| 5 | All Colorectal Cancer | rs2239619 | A | C | 0.583842 | -0.04459 | 0.021041 | 0.034063 |
| 6 | All Colorectal Cancer | rs77691416 | C | A | 0.069917 | -0.01 | 0.03992 | 0.802248 |
| 7 | All Colorectal Cancer | rs445 | T | C | 0.058233 | 0.058517 | 0.044651 | 0.190009 |
| 8 | All Colorectal Cancer | rs10103048 | C | A | 0.586765 | 0.032237 | 0.020991 | 0.124594 |
| 9 | All Colorectal Cancer | rs3087852 | A | G | 0.435189 | -0.00567 | 0.02092 | 0.786229 |
| 10 | All Colorectal Cancer | rs4134826 | C | T | 0.170614 | 0.040192 | 0.027605 | 0.145406 |
| 11 | All Colorectal Cancer | rs34861192 | A | G | 0.002931 | 0.041395 | 0.194844 | 0.831756 |
| 12 | All Colorectal Cancer | rs3745367 | A | G | 0.242717 | 0.042078 | 0.0244 | 0.084619 |
| 13 | All Colorectal Cancer | rs10401670 | C | T | 0.652247 | 0.021369 | 0.021971 | 0.330744 |
| 14 | All Colorectal Cancer | rs73008259 | A | G | 0.027612 | 0.025913 | 0.063462 | 0.683033 |
| 15 | All Colon | rs17405635 | A | G | 0.258354 | 0.022379 | 0.030051 | 0.456457 |
| 16 | All Colon | rs7589428 | G | A | 0.490289 | 0.071108 | 0.026283 | 0.00682 |
| 17 | All Colon | rs6775731 | C | T | 0.722953 | 0.025802 | 0.029497 | 0.381728 |
| 18 | All Colon | rs7746716 | T | A | 0.518445 | 0.044683 | 0.026523 | 0.092045 |
| 19 | All Colon | rs2239619 | A | C | 0.583913 | -0.05124 | 0.026854 | 0.056374 |
| 20 | All Colon | rs77691416 | C | A | 0.069938 | 0.02532 | 0.050678 | 0.617344 |
| 21 | All Colon | rs445 | T | C | 0.058207 | 0.050412 | 0.056739 | 0.374281 |
| 22 | All Colon | rs10103048 | C | A | 0.586702 | 0.024462 | 0.026752 | 0.36052 |
| 23 | All Colon | rs3087852 | A | G | 0.435203 | -0.00246 | 0.026679 | 0.926411 |
| 24 | All Colon | rs4134826 | C | T | 0.170532 | 0.017651 | 0.035211 | 0.616158 |
| 25 | All Colon | rs34861192 | A | G | 0.002931 | 0.114598 | 0.251114 | 0.648133 |
| 26 | All Colon | rs3745367 | A | G | 0.242633 | 0.031029 | 0.031099 | 0.318401 |
| 27 | All Colon | rs10401670 | C | T | 0.65222 | 0.014164 | 0.028049 | 0.613568 |
| 28 | All Colon | rs73008259 | A | G | 0.02761 | 0.07028 | 0.081034 | 0.385784 |
| 29 | All Rectal | rs17405635 | A | G | 0.258254 | -0.03765 | 0.038118 | 0.323301 |
| 30 | All Rectal | rs7589428 | G | A | 0.490162 | 0.058129 | 0.033524 | 0.082928 |
| 31 | All Rectal | rs6775731 | C | T | 0.722807 | -0.06022 | 0.037494 | 0.108275 |
| 32 | All Rectal | rs7746716 | T | A | 0.518367 | 0.031432 | 0.033609 | 0.349671 |
| 33 | All Rectal | rs2239619 | A | C | 0.584025 | -0.02235 | 0.034034 | 0.511327 |
| 34 | All Rectal | rs77691416 | C | A | 0.069846 | -0.10414 | 0.064478 | 0.106296 |
| 35 | All Rectal | rs445 | T | C | 0.058203 | 0.088227 | 0.07213 | 0.221268 |
| 36 | All Rectal | rs10103048 | C | A | 0.586771 | 0.064704 | 0.033928 | 0.056509 |
| 37 | All Rectal | rs3087852 | A | G | 0.435171 | -0.02594 | 0.033811 | 0.443038 |
| 38 | All Rectal | rs4134826 | C | T | 0.170587 | 0.086061 | 0.044697 | 0.054175 |
| 39 | All Rectal | rs34861192 | A | G | 0.002924 | -0.10842 | 0.320781 | 0.735379 |
| 40 | All Rectal | rs3745367 | A | G | 0.242635 | 0.054109 | 0.039416 | 0.169819 |
| 41 | All Rectal | rs10401670 | C | T | 0.652264 | 0.040714 | 0.03553 | 0.251833 |
| 42 | All Rectal | rs73008259 | A | G | 0.027578 | -0.02528 | 0.102615 | 0.805405 |
